# Chemical genetic activation of the cholinergic basal forebrain hippocampal circuit rescues memory loss in Alzheimer’s disease

**DOI:** 10.1186/s13195-022-00994-w

**Published:** 2022-04-13

**Authors:** Weilin Liu, Jianhong Li, Minguang Yang, Xiaohua Ke, Yaling Dai, Huawei Lin, Sinuo Wang, Lidian Chen, Jing Tao

**Affiliations:** 1 College of Rehabilitation Medicine, Fujian University of Traditional Chinese Medicine, Fuzhou, 350122 Fujian China; 2 The Academy of Rehabilitation Industry, Fujian University of Traditional Chinese Medicine, Fuzhou, 350122 Fujian China; 3 Rehabilitation Medical Technology Joint National Local Engineering Research Center, Fuzhou, 350122 Fujian China

**Keywords:** Alzheimer’s disease, Learning and memory, Chemical genetics, Cholinergic neural circuit, Neurochemical metabolism

## Abstract

**Background:**

The degeneration of the cholinergic circuit from the basal forebrain to the hippocampus contributes to memory loss in patients suffering from Alzheimer’s disease (AD). However, the internal relationships between the acetylcholine (Ach) cycle and memory decline during the early stages of AD currently remain unknown. Here, we investigate the mechanisms underlying the activation of the cholinergic circuit and its impact on learning and memory using APP/PS1 mice models.

**Methods:**

Novel object recognition and Morris water maze tests were used to measure learning and memory function. Magnetic resonance spectrum (MRS) imaging was applied to longitudinally track changes in neurochemical metabolism in APP/PS1 mice aged 2, 4, 6, and 8 months. The number of neurons and the deposition of Aβ plaques were measured using Nissl, immunohistochemistry, and Thioflavin S staining. We then employed a chemogenetic strategy to selectively activate the cholinergic circuit from the medial septal nucleus (MS) and the vertical limb of the diagonal band nucleus (VDB) on the basal forebrain to the hippocampus. MRS and immunoblotting techniques were used to measure the neurochemical metabolism levels and cholinergic-related proteins, respectively.

**Results:**

We found that the levels of choline (Cho) in the basal forebrain were markedly higher compared to other brain regions and that its decrease along with N-acetyl aspartate (NAA) levels in the hippocampus was accompanied by memory deficits in APP/PS1 mice aged 4, 6, and 8 months. In terms of pathology, we observed that the deposition of Aβ plaques gradually aggravated throughout the cerebral cortex and hippocampus in APP/PS1 mice aged 6 and 8 months, while no Aβ deposition was detected in the basal forebrain. In contrast, the activity of choline acetyltransferase (ChAT) enzyme in the basal forebrain was decreased at 6 months of age and the cholinergic neurons were lost in the basal forebrain at 8 months of age. In addition, the activation of the cholinergic circuit from the MS and VDB to the hippocampus using chemical genetics is able to improve learning and reduce memory impairment in APP/PS1 mice. Similarly, the levels of Cho in the basal forebrain; NAA in the hippocampus, as well as the expression of ChAT and vesicular acetylcholine transporter (vAchT) in the basal forebrain; and muscarinic acetylcholine receptor 2 (CHRM2) in the hippocampus all increased.

**Conclusions:**

These findings demonstrate that the neurochemical Cho and NAA of the cholinergic circuit can be used as biomarkers to enable the early diagnosis of AD. In addition, memory impairment in APP/PS1 mice can be attenuated using chemical genetics-driven Ach cycle activity of the cholinergic circuit.

**Supplementary Information:**

The online version contains supplementary material available at 10.1186/s13195-022-00994-w.

## Background

Alzheimer’s disease (AD) is a progressive and fatal neurodegenerative disease that typically occurs in people aged 65 years of age or more. AD typically manifests by a progressive loss of cognitive function and daily activity and the onset of neuropsychiatric disorders [1, 2]. The World Alzheimer Report from 2018 estimated that approximately 50 million people worldwide suffer from dementia and that this number will likely to rise to 152 million by 2050, which represents more than a 3-fold increase compared to today. In addition, it is estimated that the global societal cost for the treatment of dementia reached $1 trillion in 2018 and will rise to $2 trillion by 2030.

The degeneration of the cholinergic function is one of the main pathologies in patients suffering from AD, and the deposition of amyloid β (Aβ) plaque deposition and neurofibrillary tangles are thought to be responsible for cognitive impairment associated with the disease [3]. Autopsy reports on Alzheimer’s patients show that cholinergic neurons decrease by 70–80% in the basal forebrain, while choline acetyltransferase (ChAT) activity is reduced by 50–85% in the cerebral cortex and the hippocampus [4, 5]. The emergence of magnetic resonance imaging (MRI) technology made it possible to study brain structure and the integrity of neuro fibers and uncover atrophies in the basal forebrain and the interruption of cholinergic neurological connectivity between the basal forebrain and the hippocampus [6–8]. Over the past decades, it has been proposed that as AD advances, the degeneration of the cholinergic system is characterized by a decrease in acetylcholine (Ach), ChAT, and an enhancement of acetylcholine esterase (AchE) in the basal forebrain, cortex, and hippocampus which is known as the cholinergic hypothesis [9, 10]. Most importantly, the cholinergic system has become a therapeutic target for AD. Presently, the Food and Drug Administration (FDA) has approved 4 kinds of AchE inhibitors (tacrine, donepezil, rivastigmine, galanthamine) for the treatment of AD. However, these drugs are not effective to cure this disease and cause side effects such as nausea, vomiting, diarrhea, or anorexia [11–13]. Moreover, the use of AchE inhibitors is not recommended for the treatment of moderate AD until patients score below 20 points in the Mini-Mental State Examination (MMSE), which has a maximum of 30 points [14]. Accordingly, despite the ability of AchE inhibitors to delay cognitive decline and improve neuropsychiatric symptoms, it is nonetheless necessary to re-recognize the role played by the cholinergic system in AD progression, especially during the early stages of the disease.

The cholinergic system is a major neuromodulator of the brain and is distributed on a number of different nuclei. Among these, the medial septal nucleus (MS) and the vertical limb of the diagonal band nucleus (VDB) in the basal forebrain represent an important source of cholinergic neurons and send the majority (up to 90%) of cholinergic projections to the hippocampus to regulate episodic memory [15, 16]. Previous studies have demonstrated that acetylcholine released from the basal forebrain causes an oscillation in the theta waves by targeting the muscarinic acetylcholine receptor M (CHRM) of hippocampal pyramidal neurons, which promotes learning and memory function [17, 18]. Another study revealed that electrical or optogenetic stimulation in the MS region can activate cholinergic input in the hippocampus and enhance the synaptic plasticity of the hippocampal Schaeffer collateral pathway that promotes learning and memory in wild type mice [19]. These studies suggest that the cholinergic circuit from the MS/VDB of the basal forebrain projection to the hippocampus can modulate the processing of episodic memory. However, cholinergic circuits are impaired by neurofibrillary tangles and chemical phenotypic changes in cholinergic neurons as AD progresses [15]. Magnetic resonance spectroscopy (MRS) is a technique that quantifies the chemical changes associated with impaired neuronal integrity and cholinergic activity because of disease [20]. N-Acetyl aspartate (NAA) is a neurochemical metabolite which is detected by MRS and considered a chemical marker of neural integrity, indicating a regional decline in the hippocampus and posterior cingulate gyrus in patients suffering from AD [21]. Choline (Cho), the precursor of Ach, decreases in the basal forebrain and the hippocampus of patients with AD [22, 23]. Previously, we used animal MRI/MRS measurements and showed that NAA declines in the hippocampus in APP/PS1 mice during moderate-severe pathological stages [24], suggesting that abnormal AD changes in cholinergic metabolism impair cholinergic activity and the integrity of cholinergic neurons in the basal forebrain and hippocampus.

In light of this, it is possible to conclude that the degeneration of the cholinergic circuit from the basal forebrain to the hippocampus contributes to memory loss in AD, even though the early cholinergic activity and metabolic changes associated with memory dysfunction in AD remain poorly understood. Here, we examined memory loss improvement in APP/PS1 mice using longitudinal tracking of the early cholinergic changes in the basal forebrain and hippocampus by ^1^H MRS following memory performance evaluation and chemical genetic activation of the cholinergic circuit from MS/VDB to the hippocampus. We provide insights that deepen our understanding of cholinergic dysfunction during the early stages of AD and that can help in developing more effective therapeutic interventions to improve AD-related cognitive impairment.

## Materials and methods

### Animals and ethics

Male APPswe/PSEN1dE9 mice and female wild-type mice were purchased from the Model Animal Research Center of Nanjing University (SYXK2013-009). The animals were fed and cultured in the SPF laboratory of the Experimental Animal Center of Fujian University of Traditional Chinese Medicine. For the current experiment, we obtained a mouse line by crossing APP/PS1 males with wild type females. The animals were raised by the Experimental Animal Center of Fujian University of Traditional Chinese Medicine [license number: SYXK (Min) 2019-0007]. The mice were housed in groups of 3–5 mice/cage under a condition of 12-h light-dark cycle, consistent ambient temperature (21–25°C) and humidity (60–70%). All animal experiments were conducted following the Guidelines for Animal Experimentation of the Fujian University of Traditional Chinese Medicine approved by the Animal Experimental Ethics Committee of the Fujian University of Traditional Chinese Medicine (Fuzhou, Fujian province, China).

### Experimental protocol

This study was divided into two experiments. In experiment I, we detected the neurochemical substance alterations in APP/PS1 mice with the progression of AD. Specifically, we evaluated learning memory ability, and the neurochemical substances NAA, Cho, and mI in the basal forebrain and the hippocampus were detected in WT and AD groups at 2, 4, 6, and 8 months of age. These evaluations were accompanied by behavioral tests and MRS imaging. Each mouse group contained a total of 7 individuals. In experiment II, we chose 4-month-old APP/PS1 mice based on the results of experiment I to reveal the treatment effects of chemical genetic technology activating the cholinergic neural circuit from the MS/VDB to the hippocampus. Specifically, 4-month-old APP/PS1 mice were randomly divided into a CNO group and a Saline group, each composed of 7 individuals. Thirty days after the injection of the hM3dq virus, the mice in the CNO group were injected with a clozapine-N-oxide (CNO) solution to activate the cholinergic neural circuit, while the mice in the Saline group were injected with the same volume of saline to serve as controls. We measured the learning memory ability and neurochemical metabolism of the two groups before and after CNO/saline injection.

### New object recognition

The new object recognition (NOR) apparatus consisted of a white box (50×50×50cm). Object “A” was the same as object “a,” which were regarded as familiar objects. In addition, there was an unfamiliar object “B.” A camera was installed directly above the apparatus to record the activities of the mice, including the time each mouse explored each of these objects. Before the NOR test, each mouse was placed into the empty box to explore freely for a total of 5 min and adapt to the “new” environment. In the familiar stage, objects “A” and “a” were put in two adjacent corners of the NOR box, after which each mouse was put into the box to freely explore for 10 min. The test stage was conducted 1 or 24 h after the familiar stage. In this stage, the object “a” was replaced by the unfamiliar object “B,” after which each mouse was put into the box to freely explore for 5 min. The apparatus was wiped with 75% alcohol to eliminate the odor after each test. The time that each mouse spent exploring the familiar object (TF) and the unfamiliar object (TN) was recorded separately. The recognition index (RI) for the unfamiliar object was defined as RI = TN/(TF + TN) × 100%.

### Morris water maze test

The Morris water maze apparatus consisted of a white circular pool with a diameter of 120 cm and a height of 50cm. The water pool was divided into four equal quadrants, numbered from 1 to 4; a platform diameter of 7.5 cm was placed in the third quadrant, 2 cm under the surface of the water, which was maintained at a temperature of 24±1°C. During the positioning navigation stage, each mouse was placed into the water from four different quadrants each day. This stage lasted a total of 4 days. The time was recorded as the escape latency when the mouse found the platform within 90s and lasted for 3s. Otherwise, we recorded the escape latency as 90s and the mouse would be guided to the platform for 15s to learn. After the positioning navigation stage, we performed a space exploration test at day 5. In this stage, the platform was removed and each mouse was put into the water from the first quadrant and allowed to explore freely for 90s. All activities were recorded, including the time spent in each quadrant, the time of crossing the platform, and the swimming trajectory.

### Magnetic resonance spectroscopy

Magnetic resonance spectroscopy (MRS) was performed using a Bruker Biospec 7.0T (70/20USR MRS scanner, Bruker Biospin, Germany). The animals were anesthetized using mixed gas (1.5~2% isoflurane and 30% oxygen). The body temperature of the mice was kept constant using a circulating hot water pump, while their respiratory frequency was constantly monitored. We used location imaging to ensure the head of each mouse was at the center of the image and then employed T2WI images to determine the volumes of interest (VOI). The basal forebrain and the hippocampus were selected as the VOI for scanning with the following parameters: TR=1500ms and TE=144ms. The positions of the neurochemical substances were shown as NAA 2.02 parts per million (ppm), Cho 3.20 ppm, mI 3.60ppm, and Cr 3.05 ppm. After data collection, we used the software package TOPSPIN (v3.1, Bruker Biospin, Germany) to process the spectral data. A quantum estimation (QUEST) method was used to calculate the ratio of the area under the curve of NAA and Cr (NAA/Cr), which represents the chemical metabolic levels of NAA. The chemical metabolic levels of Cho (Cho/Cr) and mI (mI/Cr) were calculated in the same fashion.

### Designer receptors exclusively activated by designer drugs (DREADDs)

In experiment II, we injected adeno-associated virus encoding hM3Dq into the MS and VDB nuclei to selectively express the hM3Dq receptors in their respective cholinergic neurons. Detailed procedures are described below. The mice in the CNO and Saline groups were anesthetized with 1% pentobarbital sodium (0.05 g/kg i.p.) and then positioned in a stereotaxic apparatus (RWD, Shenzhen, China). The rAAV-ChAT-cre-2a-eGFP-WPRE-pA and rAAV-ef1a-DIO-hM3Dq-mCherry-WPRE-pA (1×10^12^ particles/ml, Wuhan Institute of Physics and Mathematics, Chinese Academy of Sciences) viruses were mixed in a ratio of 1:3. The mixed virus solution was injected (400nl) into the MS/VDB region (AP +1.10 mm, ML 0.00 mm, DV −4.80 mm; relative to the bregma; AP, ML, and DV denote anteroposterior, mediolateral, and dorsoventral distance from the bregma, respectively) for 10 min using a 10-μl microsyringe. All injections were followed by an additional 5 min to allow for the virus to diffuse before removing the microsyringe. One month after injecting the virus, the clozapine N-oxide solution (0.33 mg/ml dissolved in saline) was administered intraperitoneally 30 min before behavioral tests and MRS imaging were performed in the CNO group (0.2 ml/20g). The same volume of saline was administered to the Saline group.

### Thioflavin and Nissl staining

In experiment I, we detected pathological changes in the basal forebrain and hippocampus in 6- and 8-month-old APP/PS1 mice. The WT and AD mice groups were anesthetized with 1% pentobarbital sodium (0.05 g/kg i.p.). Saline and 4% paraformaldehyde were perfused from the left ventricle to, respectively, wash and fix the brain tissue. Then coronal slices of the brain tissue were prepared for pathological staining, using 5μm per section. To perform Thioflavin S staining, the tissues were placed in 0.3% Thioflavin S solution (Thioflavin S, Sigma, T1892) and then differentiated in 50% ethanol. Nissl staining was performed with Nissl Staining Solution (Nissl Staining Solution (Cresyl Violet), Solarbio, G3410) according to the manufacturer’s instructions.

### Immunohistochemistry staining

Immunohistochemical staining was performed by standard methods. Paraffin-embedded tissues were sliced into 5-μm sections. Citric acid buffer was used at 95°C for 1–2 h for antigen retrieval. Then the sections were incubated in 0.3% H_2_O_2_ for 10 min. Following this procedure, the sections were washed in PBS for 10 min and then blocked in 3% bovine serum albumin (BSA) for another 30 min. Next, the sections were incubated with primary antibody goat-anti-ChAT (Sigma, AB144P, 1:100) for 48 h at 4 °C. And then the sections were washed in PBS for 10 min and incubated with secondary antibody biotinylated-anti-goat (Vector Laboratories, PK4005, 1:100) for 2 h. Then the brain sections were washed in PBS for 5 min and incubated with ABC-kit solution (Vector Laboratories, PK-4005) for 2 h. After being washed in PBS for 10 min, the brain sections were stained in DAB staining solution for 3 min and then quickly washed in PBS. Finally, the sections were mounted with neutral resin. The average number of ChAT-positive neurons in the basal forebrain was calculated manually and the average optical density value was calculated by ImageJ.

### Western blotting

In experiment II, cholinergic-related proteins of the basal forebrain and hippocampus in the CNO and Saline groups were detected through Western blotting after DREADDs intervention. The brain tissues of the basal forebrain and hippocampus were readily put on ice. RIPA lysate was used to extract the protein, and a BCA assay was then applied for protein quantification. Brain homogenates were resolved by SDS–polyacrylamide gel electrophoresis (SDS-PAGE) and transferred to activated PVDF membranes. The membranes were blocked with 5% skimmed milk powder in pure water. The following antibodies were incubated overnight at 4°C: ChAT (1:5000; Abcam, 181023), AchE (1:1000; Abcam, 183591), ChT1 (1:5000; Abcam, 154186), vAchT (1:1000; Sigma, SAB4200559), CHRM2 (1:5000; Abcam, 109226), and GAPDH (1:8000; Lab, G0100). The membranes were washed in TBST and incubated with an HRP-conjugated secondary antibody (1:5000) for 1h at room temperature. The protein bands were visualized using enhanced chemiluminescence and imaged with a Bio-Image Analysis system (Bio-Rad Laboratories, Inc.).

### Verification of MS/VDB to the hippocampus circuit and expression of hM3Dq

In experiment II, the mice in the CNO and Saline groups were sacrificed after behavioral tests were performed. We observed histological verification of the MS/VDB-HIP circuit and evaluated the expression of hM3Dq receptors. The mice in the CNO and Saline groups were anesthetized with 1% pentobarbital sodium (0.05 g/kg i.p.). Saline and 4% paraformaldehyde were perfused from the left ventricle to, respectively, wash and fix the brain tissue. We then prepared coronal slices (30μm per slice) of the brain tissue for confocal laser imaging.

### Statistical analysis

The data were analyzed using the SPSS22.0 software, and the results expressed as the mean±SEM. The escape latency data acquired in the Morris water maze were analyzed by repeated measurements of variance analysis, and others were analyzed using Student *t*-tests. The difference was considered as significant when *P* < 0.05.

## Results

### Longitudinal tracking of early memory impairment processes in APP/PS1 mice

**Fig. 1:**
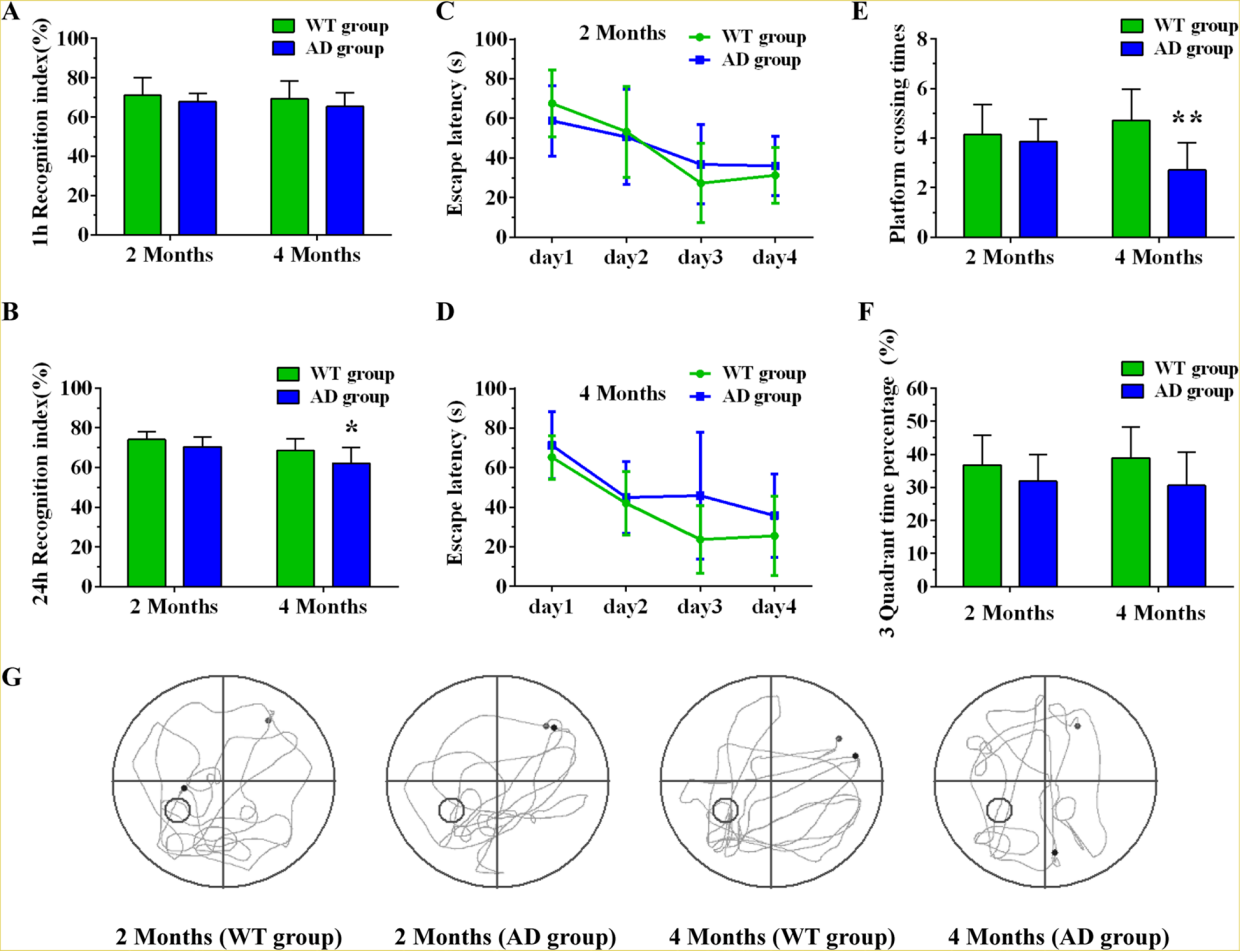
Learning and memory ability of WT and AD groups at the 2nd and 4th month as measured by NOR and Morris water maze experiments. **A**, **B** The 1-h and 24-h recognition index (RI) of WT and AD groups at the 2nd and 4th month in the NOR test. **C**, **D** The escape latency of the WT and AD groups at the 2nd and 4th month in the Morris water maze test. **E**, **F** Platform crossing times and third quadrant time percentage of WT and AD groups at the 2nd and 4th month in the Morris water maze test. **G** The trajectory diagram of WT and AD groups at the 2nd and 4th month in the Morris water maze test. *P<0.05, **P<0.01

**Fig. 2:**
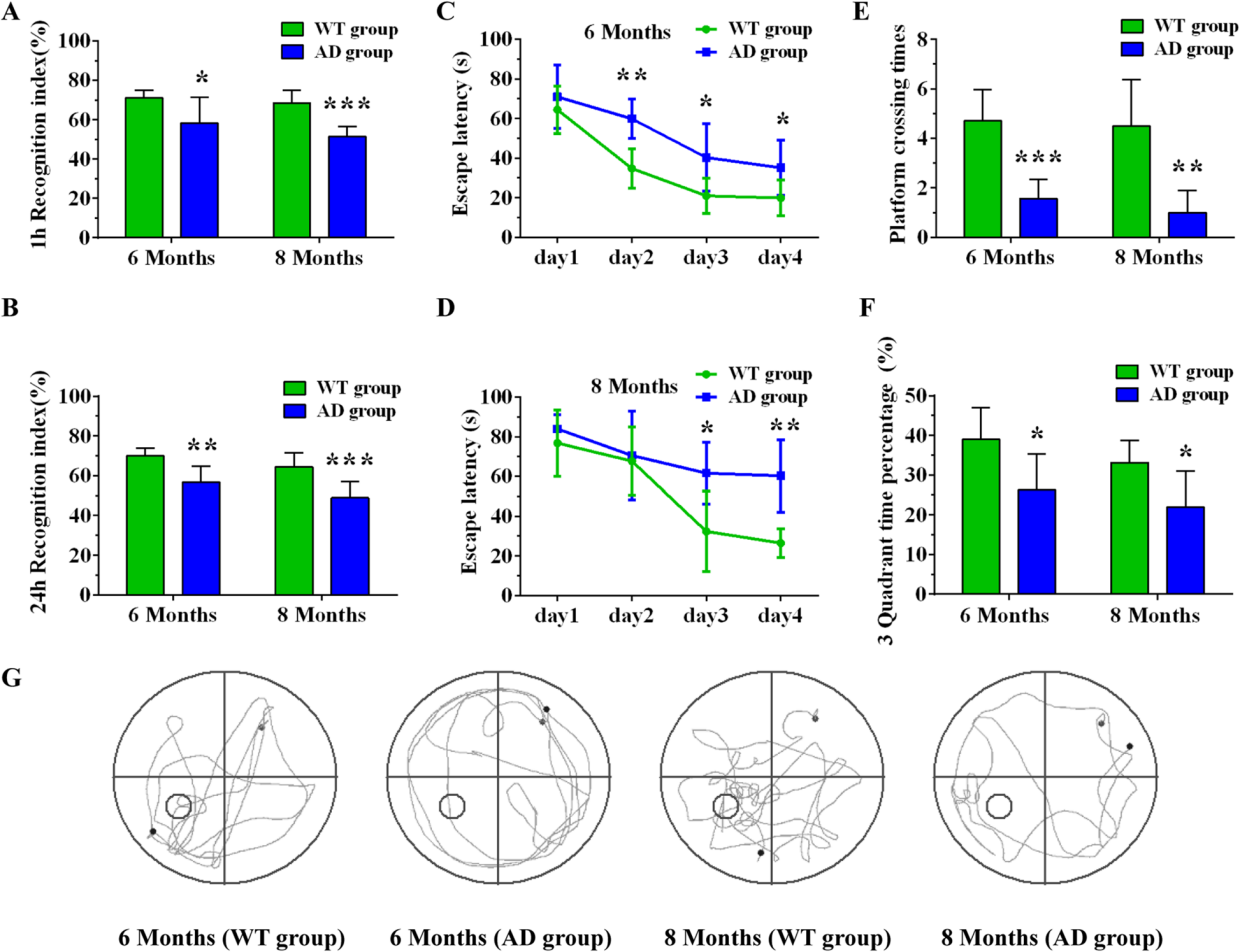
Learning and memory ability of WT and AD groups at the 6th and 8th month as measured by NOR and Morris water maze experiments. **A**, **B** The 1-h and 24-h recognition index (RI) of WT and AD groups at the 6th and 8th month in the NOR test. **C**, **D** The escape latency of the WT and AD groups at the 6th and 8th month in the Morris water maze test. **E**, **F** Platform crossing times and third quadrant time percentage of WT and AD groups at the 6th and 8th month in the Morris water maze test. **G** The trajectory diagram of WT and AD groups at the 6th and 8th month in the Morris water maze test. *P<0.05, **P<0.01, ***P<0.001

The NOR and Morris water maze tests were used to evaluate object recognition memory and spatial learning memory at the 2nd, 4th, 6th, and 8th month, respectively. The results of the NOR test showed the recognition index gradually decreased with age in AD mice. Specifically, we found no significant difference between the two groups on the 1-h and 24-h recognition index (RI) at the 2nd month (Fig. 1A, B). However, 4-, 6-, and 8-month-old AD mice showed a decreased recognition index in comparison to age-matched wild type mice (Figs. 1B and 2A, B). In regard to the positioning navigation trial in the Morris water maze, repeated-measures ANOVA showed that the escape latency of the two groups on the 2nd, 4th, 6th, and 8th month displayed a downward trend as training time increased. Comparisons between the groups showed that 6- and 8-month-old AD mice exhibited significantly longer escape latencies compared to wild type mice of the same age (Figs. 1C, D and 2C, D). In the space exploration test, we observed that both platform crossing time and percentage of time spent in the 3rd quadrant decreased in 4-, 6-, and 8-month-old mice compared with wild type mice of the same age. No changes were observed between the two groups on the 2nd month (Figs. 1E–G and 2E–G). These results suggest that the learning memory of AD mice is already impaired at the 4th month and intensifies as the disease progresses.

### Longitudinal tracking of early neurochemical metabolic abnormalities of the cholinergic circuit in APP/PS1 mice

**Fig. 3:**
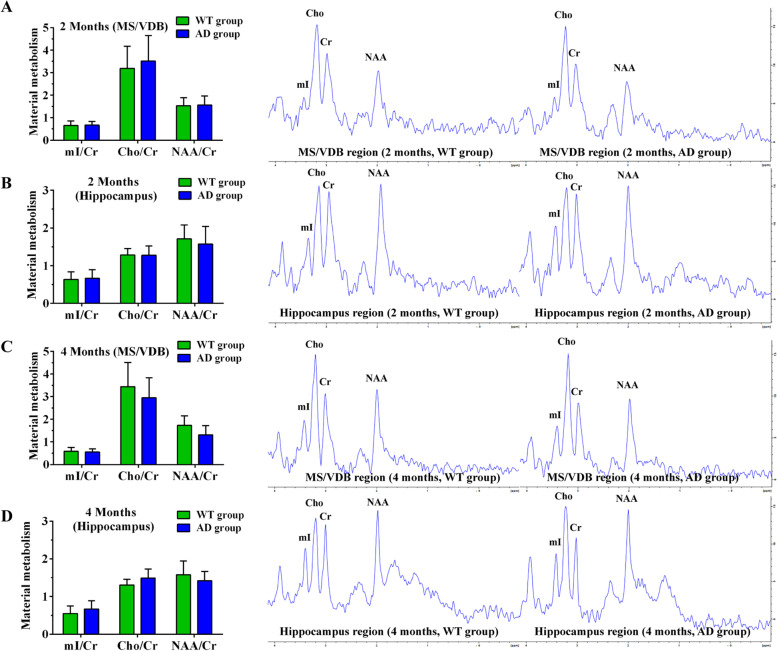
Material metabolism of WT and AD groups at the 2nd and 4th month as measured by ^1^H-MRS. **A**, **B** Material metabolism in the basal forebrain (**A**) and hippocampus (**B**) of WT and AD groups at the 2nd month. **C**, **D** Material metabolism in the basal forebrain (**C**) and hippocampus (**D**) of WT and AD groups at the 4th month

**Fig. 4:**
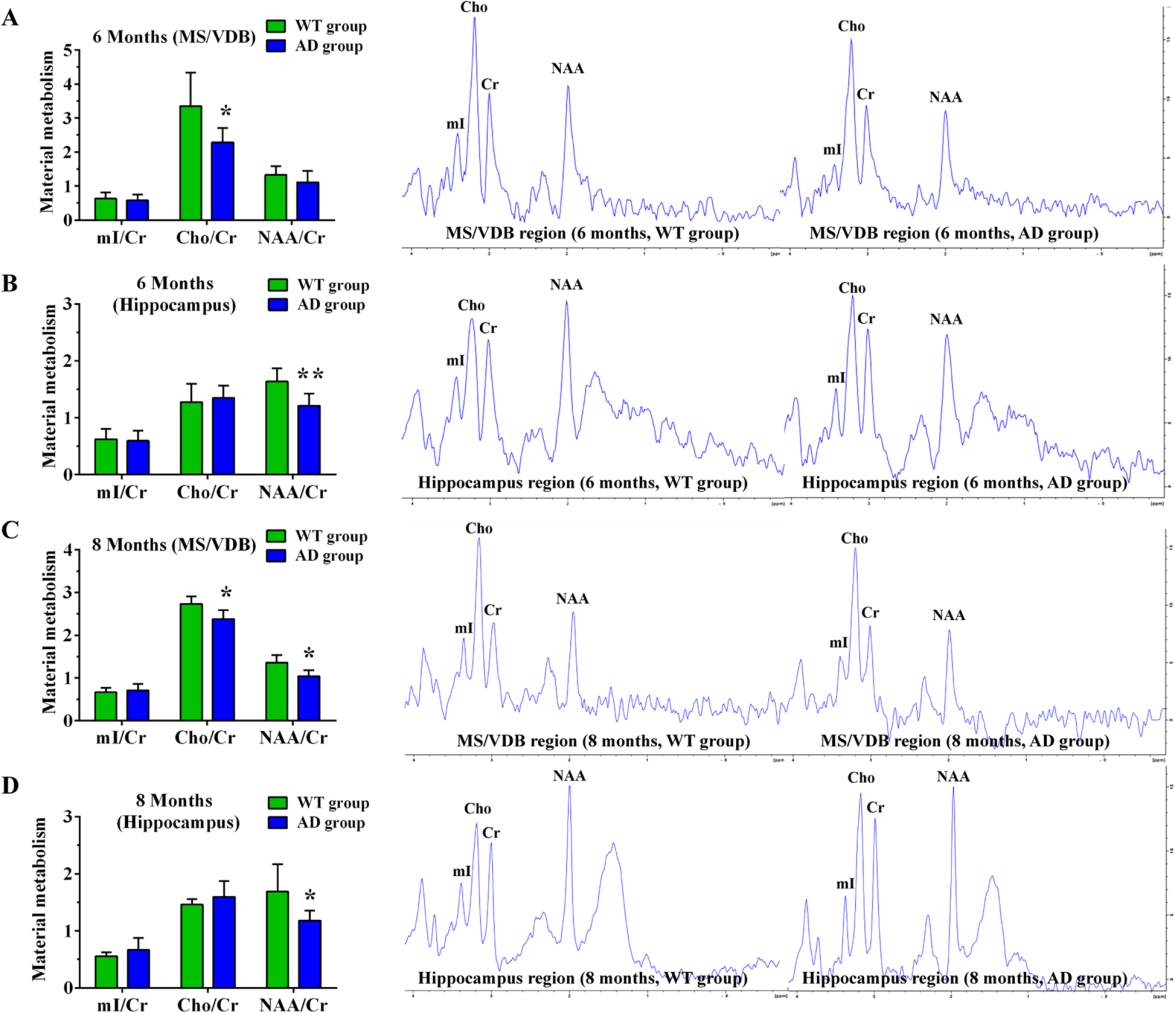
Material metabolism of WT and AD groups at the 6th and 8th month as measured by ^1^H-MRS. **A**, **B** Material metabolism in the basal forebrain (**A**) and hippocampus (**B**) of WT and AD groups at the 6th month. **C**, **D** Material metabolism in the basal forebrain (**C**) and hippocampus (**D**) of WT and AD groups at the 8th month. *P<0.05, **P<0.01

The VOI of MS and VDB nuclei of the basal forebrain and hippocampus were selected to examine ^1^H-MRS imaging in WT and AD mice groups at different ages. In ^1^H-MRS, NAA is regarded a marker of neuronal integrity while Cho is a marker of membrane synthesis and degradation and is also a potential marker for cholinergic neuron. The ^1^H-MRS results showed that the ratio of Cho/Cr in the basal forebrain is significantly higher than that of the hippocampus due to the presence of abundant cholinergic neurons in the basal forebrain (Figs. 3 and 4). A comparison between the WT and AD groups revealed the ratio of Cho/Cr in the basal forebrain decreased in 6- and 8-month-old APP/PS1 mice compared to wild types of the same age (Fig. 4A, C). We observed no difference between the two groups in the Cho/Cr ratio in the hippocampus at different ages (Figs. 3B, D and 4B, D). In contrast, the NAA/Cr ratio in the hippocampus declined at the 6th and 8th months in APP/PS1 mice compared to wild type individuals, while in the basal forebrain this decrease was only evident in 8-month-old APP/PS1 mice (Fig. 4). No changes of mI/Cr in basal forebrain and hippocampus were detected in APP/PS1 mice (Figs. 3 and 4).

### Aβ deposition in the brain and neuronal loss in APP/PS1 mice

**Fig. 5:**
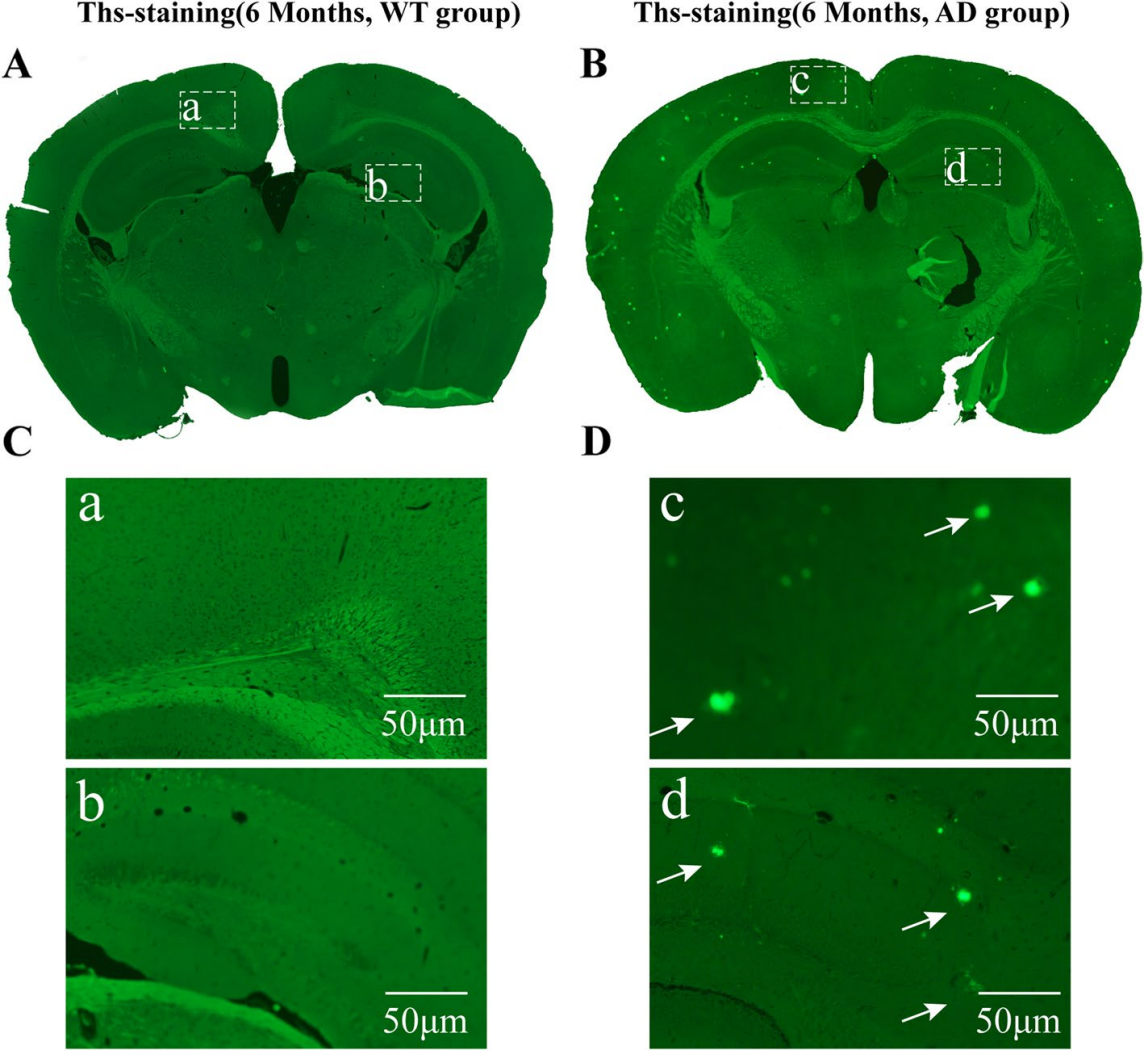
The deposition of Aβ plaques in APP/PS1 mice at the 6th month. **A**, **B** The deposition of Aβ in wild type (**A**) and APP/PS1 mice (**B**) at the 6th month as detected by Ths staining and the boxed areas are shown below. **C**, **D** The higher magnification image of Aβ plaques in wild type (**C**) and APP/PS1 mice (**D**)

**Fig. 6:**
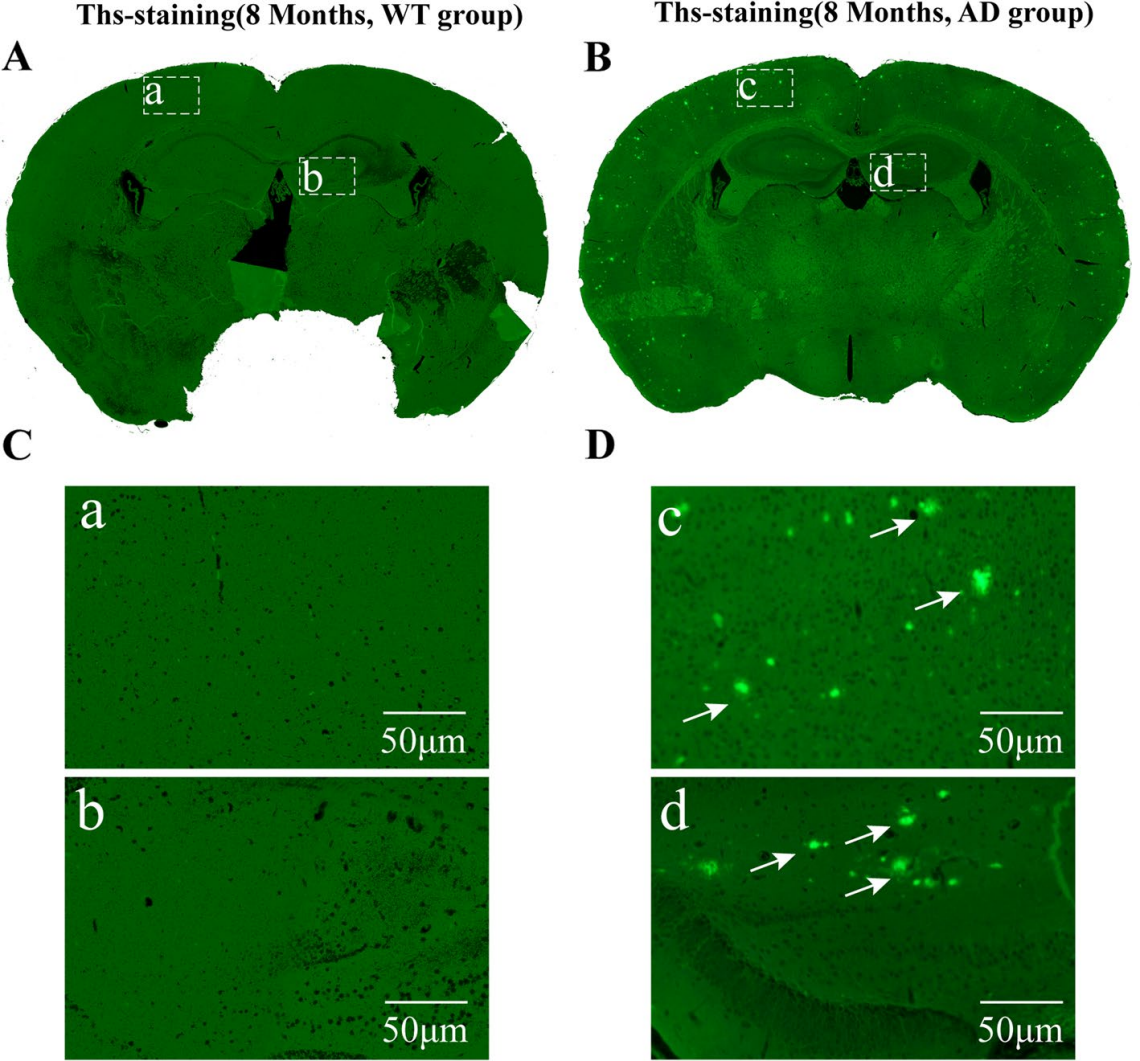
The deposition of Aβ plaques in APP/PS1 mice at the 8th month. **A**, **B** The deposition of Aβ in wild type (**A**) and APP/PS1 mice (**B**) at the 8th month as detected by Ths staining and the boxed areas are shown below. **C**, **D** The higher magnification image of Aβ plaques in wild type (**C**) and APP/PS1 mice (**D**)

**Fig. 7:**
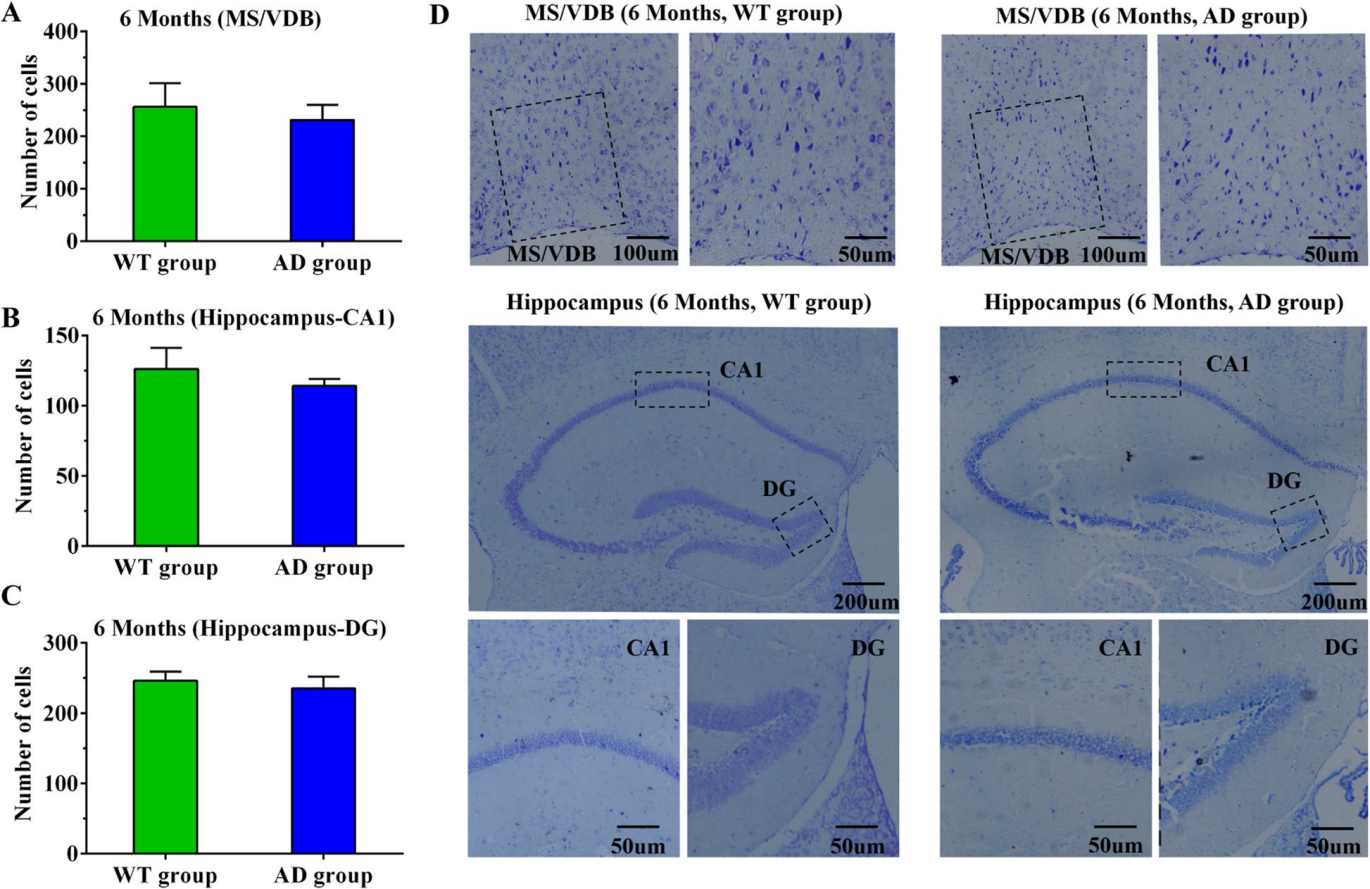
Neuron loss in the basal forebrain and hippocampus in WT and AD groups at the 6th month as detected by Nissl staining. **A** Nissl staining results in the basal forebrain of WT and AD groups at the 6th month. **B** Nissl staining results of the CA1 region of WT and AD groups at the 6th month. **C** Nissl staining results of the DG region of WT and AD groups at the 6th month. **D** Nissl staining images of related brain regions in WT and AD groups at the 6th month

**Fig. 8:**
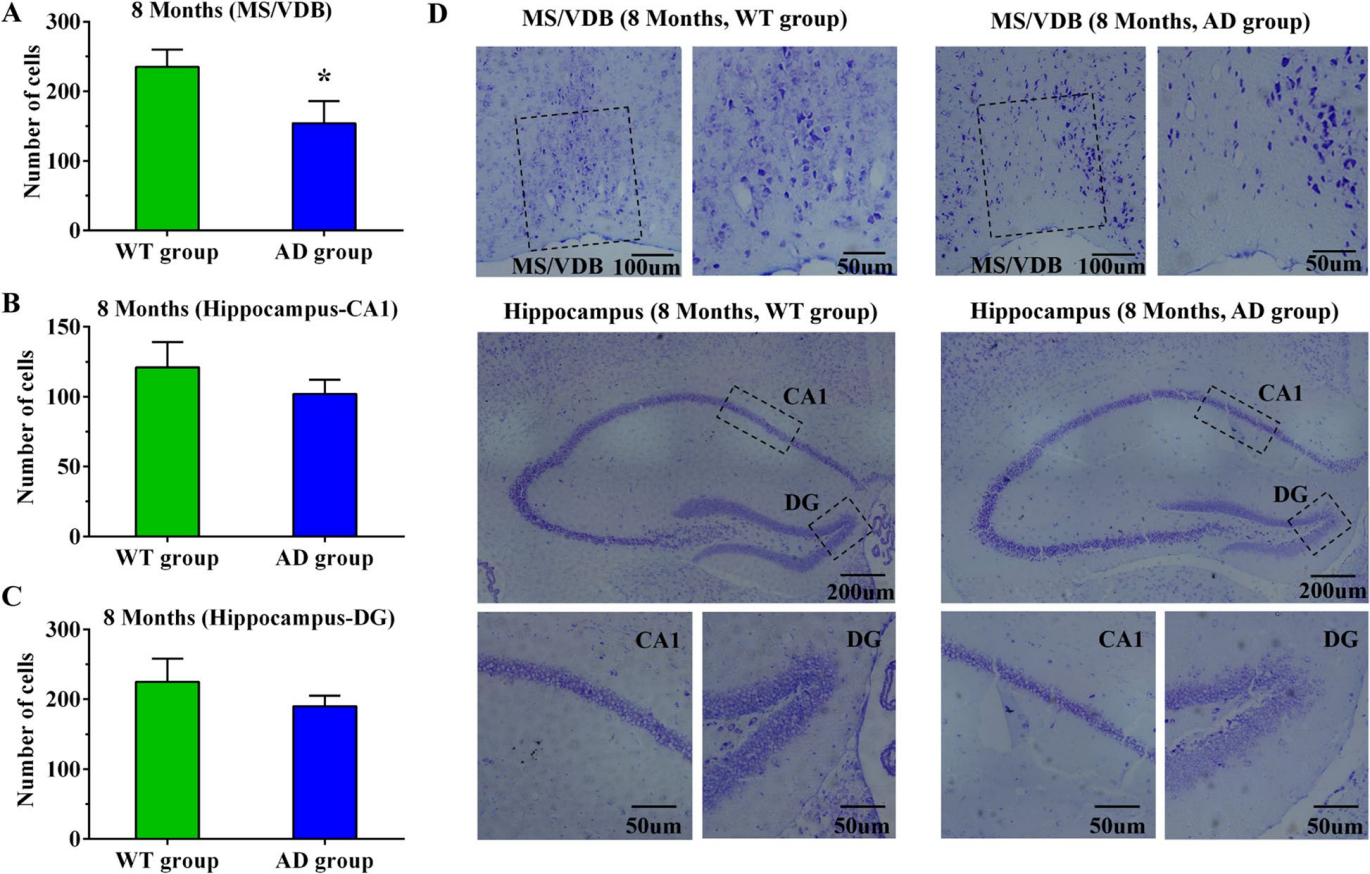
Neuron loss of basal forebrain and hippocampus in WT and AD groups at the 8th month detected by Nissl staining. **A** Nissl staining results in the basal forebrain of WT and AD groups at the 8th month. **B** Nissl staining results of the CA1 region of WT and AD groups at the 8th month. **C** Nissl staining results of the DG region of WT and AD groups at the 8th month. **D** Nissl staining images of related brain regions in WT and AD groups at the 8th month. *P<0.05

**Fig. 9:**
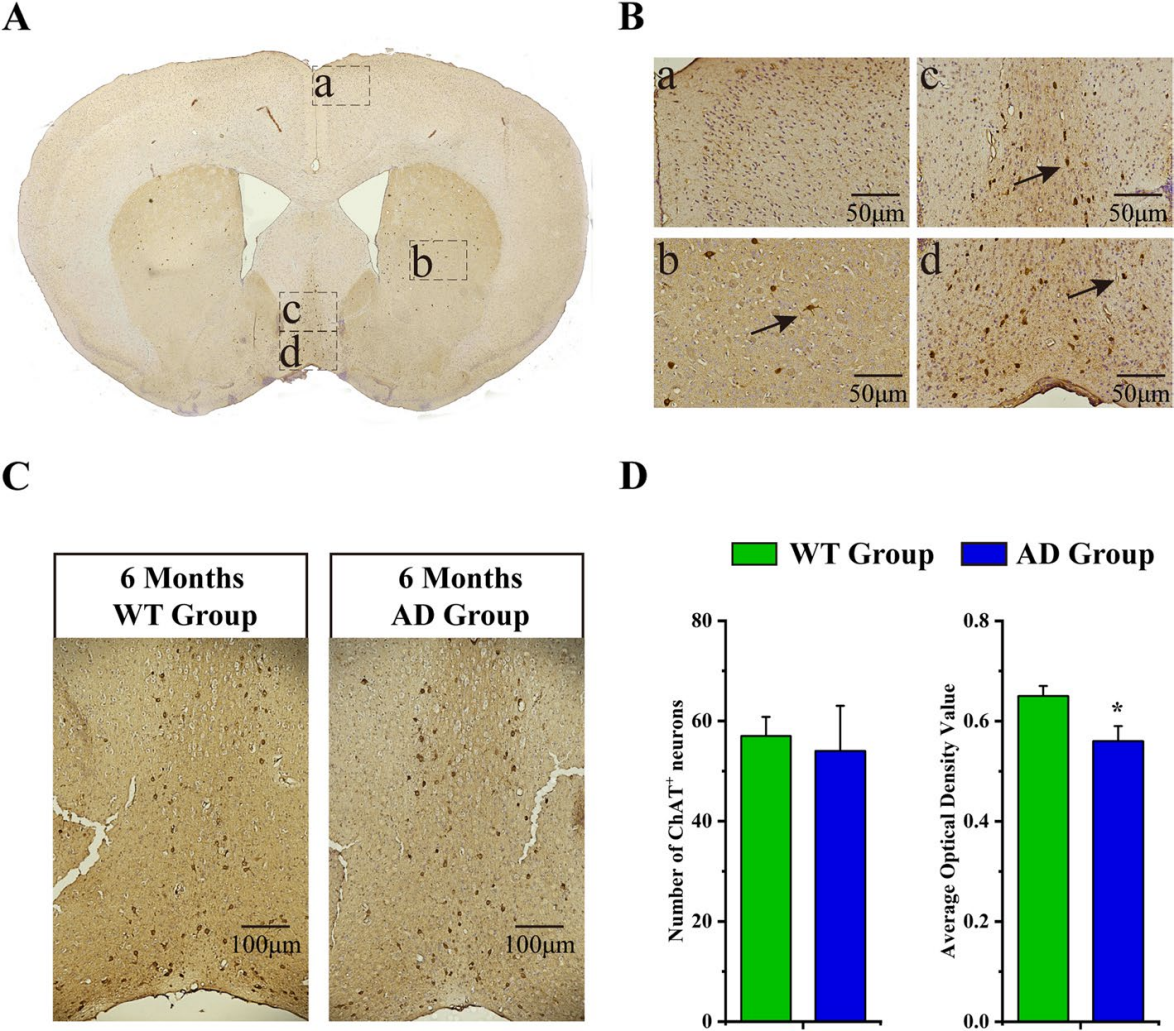
Distribution of cholinergic neurons in the mouse brain and the ChAT-positive neurons in the MS and VDB nuclei. **A** Distribution of cholinergic neurons in the mouse brain and the boxed areas are shown in right. **B** The higher magnification image of the cholinergic neurons in the cortex (a), caudate putamen (b), MS nucleus (c), and VDB nucleus (d) brain areas. **C**, **D** The number of ChAT-positive neurons and the average optical density value of ChAT in the MS and VDB nuclei of WT and AD groups at the 6th month. *P < 0.05

The pathological changes of the basal forebrain and hippocampus in APP/PS1 mice were observed using Thioflavin, Nissl staining, and immunohistochemistry staining. Thioflavin staining showed that Aβ-immunoreactive plaques were distributed throughout the cerebral cortex and the hippocampus in 6-month-old APP/PS1 mice and that Aβ plaque deposition aggravated at 8 months of age (Figs. 5 and 6). However, there was no Aβ plaque deposition in the basal forebrain in 6-, 8- and even 13-month-old APP/PS1 mice (Fig. S1). Nonetheless, Nissl staining showed that neurons in the basal forebrain were sparsely arranged in 6-month-old APP/PS1 mice and that their number decreased in individuals 8 months of age compared to wild types. No significant neuron reduction was found in the hippocampus of 6- and 8-month-old APP/PS1 mice (Figs. 7 and 8). The results of immunohistochemistry staining showed that ChAT-positive neurons are mainly located in the forebrain structures, including caudate putamen, MS nucleus, and VDB nucleus (Fig. 9A, B). And no significant ChAT-positive neuron reduction was found in the MS and VDB nuclei of 6-month-old APP/PS1 mice. However, comparisons between the WT group and AD group showed that 6-month-old AD mice exhibited significantly decreased ChAT activity in MS and VDB nuclei (Fig. 9C, D).

### Chemical genetics stimulation of the cholinergic circuit and early memory deficits in APP/PS1 mice

**Fig. 10:**
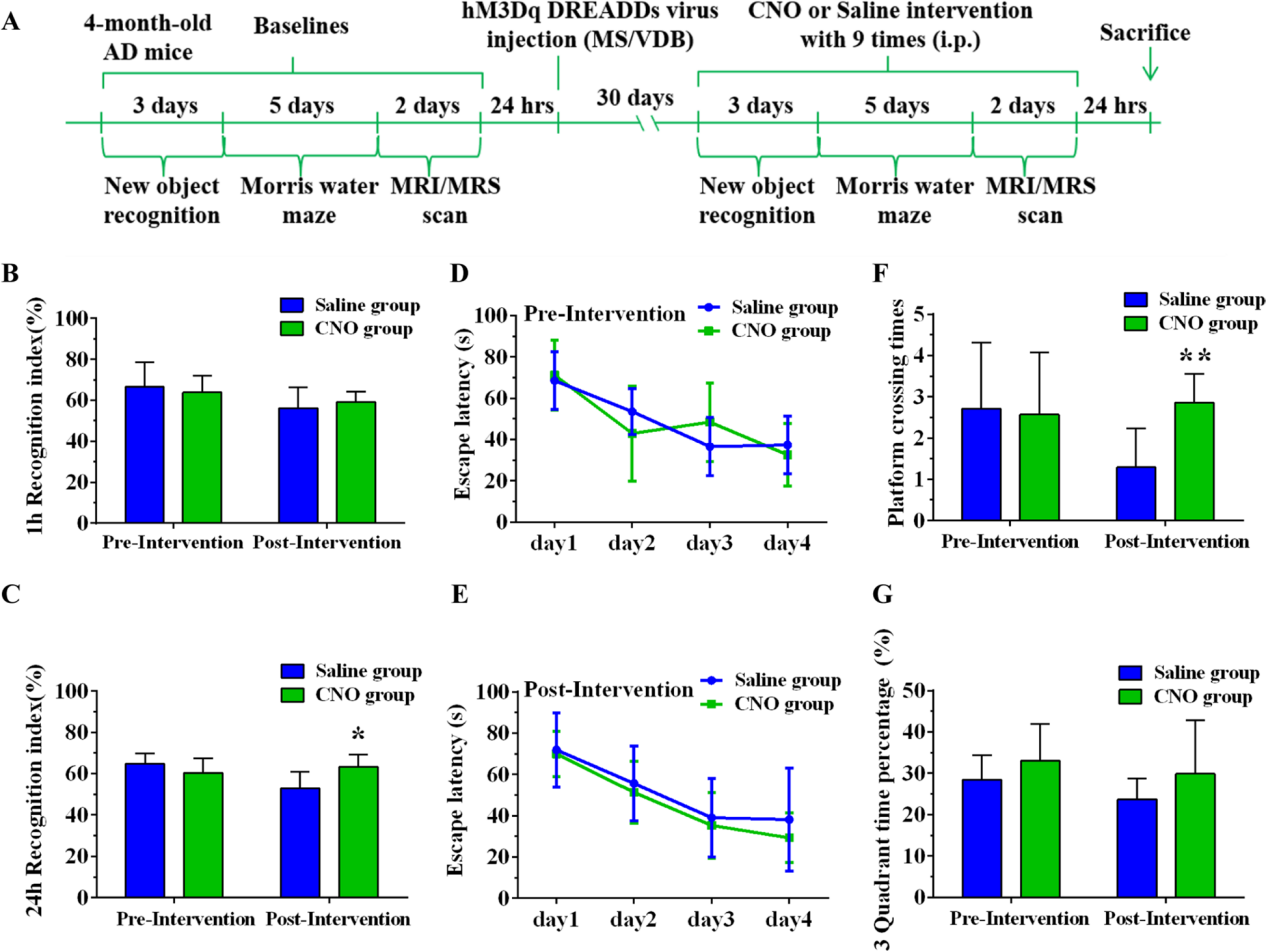
DREADDs intervention might improve learning and memory in APP/PS1 mice. **A** DREADDs intervention timeline. **B**, **C** The 1-h and 24-h recognition index (RI) of the CNO and Saline groups before and after the intervention. **D**, **E** The escape latency of the CNO and Saline groups before and after the intervention. **F**, **G** Platform crossing times and third quadrant time percentage of the CNO and Saline groups before and after the intervention. *P<0.05, **P<0.01

The results of experiment I indicated that learning memory and the function of cholinergic neural circuit were impaired in APP/PS1 mice aged 4–6 months. We therefore selected 4-month-old APP/PS1 mice for chemical genetic intervention (Fig. 10A). NOR and Morris water maze tests were used to examine the cognitive function of the CNO and Saline groups before and after chemical genetic intervention. No significant differences were found between the two groups in pre-intervention object recognition memory and spatial learning memory. Overall, chemical genetic intervention was able to ameliorate cognitive decline in the AD mouse model. Specifically, in the NOR test, we observed that the 24-h recognition index in the CNO group increased compared to the Saline group after chemical genetic intervention. In the Morris water maze test, platform crossing times in the CNO group increased compared to the Saline group (Fig. 10B–G). To further confirm the effects of chemical genetic manipulation, we increased the times of chemical genetic intervention and found a magnification of the effects of chemical genetic intervention in both NOR and Morris water maze tests (Fig. S2). These results suggest the activation of the cholinergic neural circuit from MS/VDB to the hippocampus by chemical genetic technology might improve learning and memory abilities in APP/PS1 mice.

### Chemical genetic stimulation of the cholinergic circuit for treatment of early AD in APP/PS1 mice

**Fig. 11:**
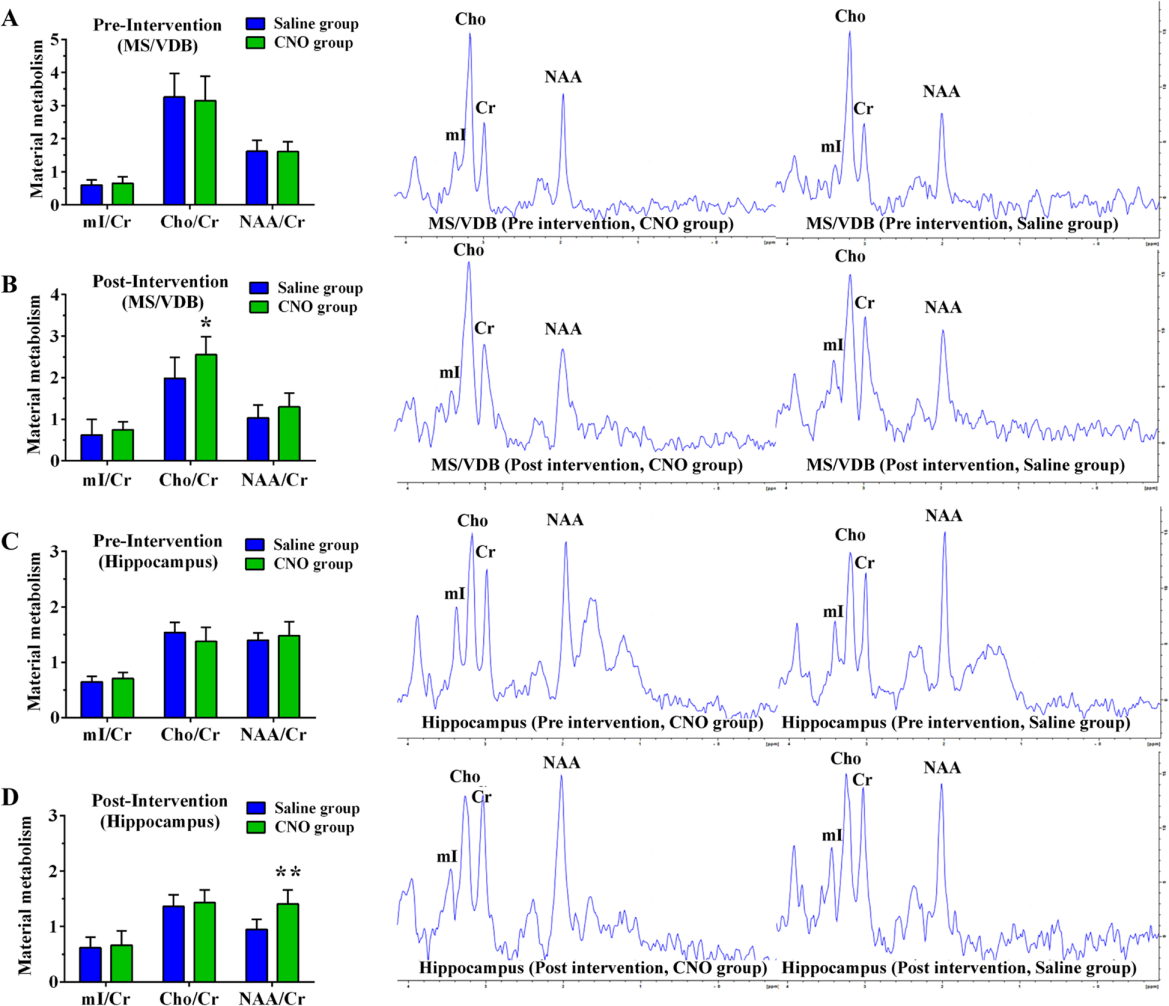
Material metabolism as measured by ^1^H-MRS in the CNO and Saline groups before and after the intervention. **A**, **B** Material metabolism in the basal forebrain of the CNO and Saline groups before and after the intervention. **C**, **D** Material metabolism in the hippocampus of the CNO and Saline groups before and after intervention

The neurochemical substances in the basal forebrain and hippocampus were measured by ^1^H-MRS before and after chemical genetic intervention. The results showed no significant differences in the ratio of Cho/Cr and NAA/Cr in the basal forebrain and hippocampus between the CNO and Saline groups before chemical genetic intervention (Fig. 11A, C). However, as shown in Fig. 11B, D, the ratio of Cho/Cr in the basal forebrain and NAA/Cr in the hippocampus increased after chemical genetic intervention. This suggests the activation of the cholinergic neural circuit from the MS/VDB to the hippocampus by chemical genetic technology might promote Cho and NAA synthesis in APP/PS1 mice.

**Fig. 12:**
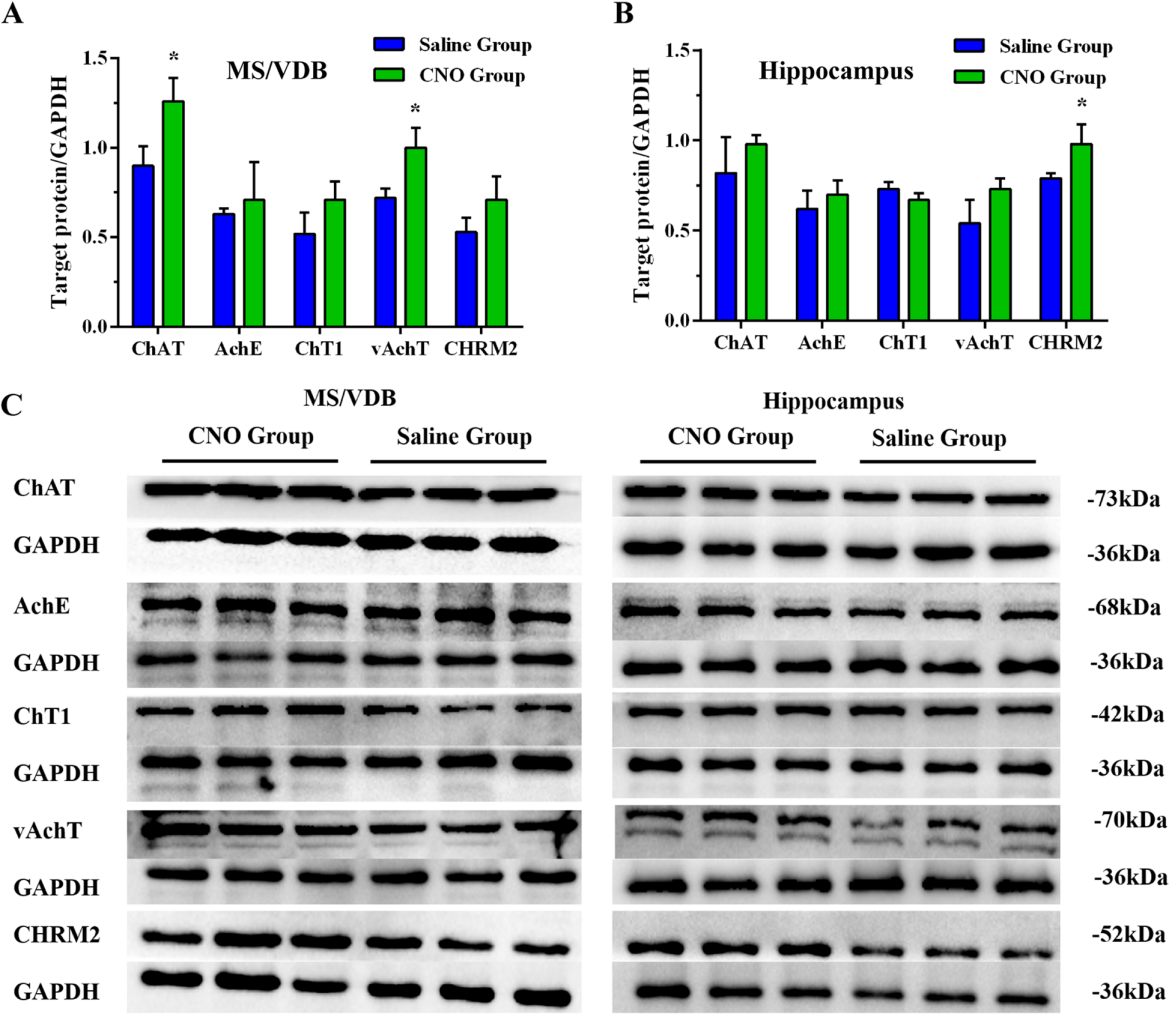
DREADDs intervention might increase the expression of cholinergic-related proteins in APP/PS1 mice. **A** The expression of cholinergic-related proteins in the basal forebrain of the CNO and Saline groups after intervention. **B** The expression of cholinergic-related proteins in the hippocampus of the CNO and Saline groups after intervention. **C** The molecular weight of each cholinergic-related protein. *P<0.05

Furthermore, the detection of cholinergic-related proteins in the basal forebrain and hippocampus confirms the function of the cholinergic neural circuit from the MS/VDB to the hippocampus after chemical genetic intervention. Western blotting showed that the CNO group exhibited significant increases in the amount of choline acetyltransferase (ChAT) and vesicular acetylcholine transporter (vAchT) in the basal forebrain after chemical genetic intervention. However, we found no significant differences in the expression of acetylcholinesterase (AchE), ChT1, and muscarinic acetylcholine receptor 2 (CHRM2) in the basal forebrain when comparing the CNO and Saline groups after chemical genetic intervention (Fig. 12A). A comparison of the hippocampus between these two groups further revealed that the expression of muscarinic receptor 2 (CHRM2) was enhanced in the CNO group following chemical genetic intervention. However, no significant differences in the expression of ChAT, AchE, ChT1, and vAchT were found between the groups after chemical genetic intervention (Fig. 12B).

### Validation of the cholinergic projection from MS/VDB to the hippocampus

**Fig. 13:**
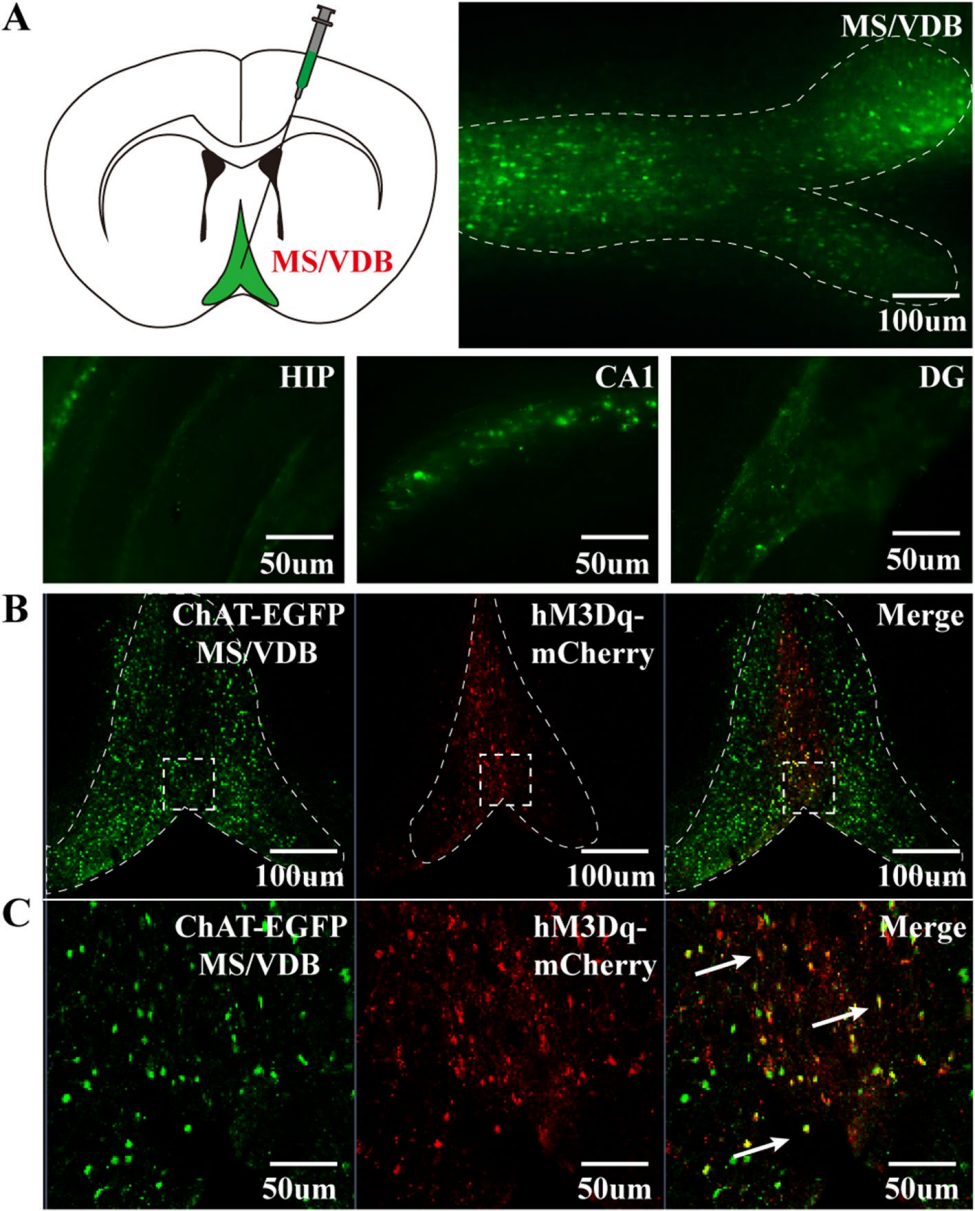
Validation of the MS/VDB-HIP circuit and expression levels of hM3dq receptors. **A** The cholinergic neurons in the MS/VDB projected to the hippocampus (green). **B**, **C** AAV virus-transfected hM3Dq receptor was successfully expressed on cholinergic neurons in the basal forebrain (yellow, as the arrows show)

We found that the recombinant adeno-associated virus with ChAT promoter-eGFP was expressed in the nuclei of MS/VDB and projected to the hippocampus (Fig. 13A), which demonstrates this region of the brain receives cholinergic projections from the basal forebrain, mainly stemming from the nuclei of MS and VDB.

The recombinant adeno-associated virus expressing hM3Dq-mCherry colocalized with ChAT-eGFP in the nuclei of MS and VDB in the basal forebrain (Fig. 13B, C), suggesting the chemical genetic excitatory receptor hM3Dq was successfully expressed in cholinergic neurons of the basal forebrain.

## Discussion

The cholinergic hypothesis was first put forward more than 30 years ago and assumes that memory deficits of AD are associated with an irreversible deficiency in cholinergic function [10]. Abundant evidence from previous studies showed that the basal forebrain neuronal projections are important for ensuring cognitive functions, such as selective attention, conscious awareness, sensory processing, and learning memory [25, 26]. In addition, it is well established that the cholinergic projection which arises from the MS and VDB nuclei of the basal forebrain to the hippocampus is associated with learning and memory ability [27–30] and that this cholinergic neural circuit is damaged as a result of AD [31]. However, currently available cholinesterase inhibitors, such as donepezil, galantamine, rivastigmine, or tacrine, which were developed following the cholinergic hypothesis, are only able to slightly reduce the short-term cognitive decline in patients suffering from AD and have high withdrawal rates due to adverse effects [32]. The powerful regulation of specific neurons by chemical genetic technology has been widely utilized to substantiate the involvement of physiological mechanisms in behavioral outcomes [33, 34]. To the best of our knowledge, our current study is the first to perform chemical genetic technology in the cholinergic neural circuit to stimulate the release of acetylcholine in APP/PS1 mice. This approach differs from the use of cholinesterase inhibitors which slow down the degradation of acetylcholine by inhibiting the activity of acetylcholinesterase. Firstly, we used MRS longitudinal tracking of neurochemical substances to reveal the metabolic changes in the MS and VDB nuclei of the basal forebrain and hippocampus in APP/PS1 mice. Surprisingly, the peak of Cho compounds in the basal forebrain was significantly higher than those in the hippocampus and other brain regions (data not shown), which makes this a potential marker of cholinergic neurons that are present in the basal forebrain. The levels of Cho decreased with the degeneration and loss of basal forebrain cholinergic neurons. Furthermore, we found that the activation of cholinergic basal forebrain neurons in MS and VDB nuclei by chemical genetics is capable of increasing Cho in the basal forebrain and is accompanied by an improvement in learning and memory behavior. Therefore, our study demonstrates that the neurochemicals Cho and NAA of the cholinergic circuit might be used as biomarkers for early AD diagnosis.

Magnetic resonance spectroscopy (MRS) is a useful neuroimaging tool to evaluate changes in neurochemical metabolism during AD. The peak in the amount of Cho shown in the MRS mainly results from the presence of glycerophosphocholine and phosphocholine in the cytoplasm. Previous studies often attributed variations in Cho peaks to neuronal integrity and membrane turnover [35] and found that the amount of Cho in the posterior cingulate and inferior precunei increased in patients suffering from AD compared to healthy individuals [36–39]. In addition, another study found that abnormal elevations in Cho/Cr ratio were associated with poorer performances in cognition tests [40] and that people with a higher Cho/Cr ratio in the white matter above the lateral ventricles had a higher risk to develop Alzheimer’s disease within the following 4 years [41]. However, other studies reached contrasting conclusions. For example, a study used MRS to measure the Cho/Cr ratio in the brain and found it was decreased in several brain regions in patients with AD, including the posterior cingulate, thalamus, frontotemporal areas, and basal ganglia [23]. A different study found the Cho/Cr ratio decreased in the hippocampus and posterior cingulate in patients suffering from AD [42]. Combined with MRS and ^18^F-fluoro-2-deoxy-d-glucose positron emission tomography (FDG-PET), decreases in the Cho/Cr ratio have been observed in the supraventricular white matter and the medial cortex of patients with AD. Further analysis found the level of Cho/Cr correlates with cognitive status and cerebral glucose metabolism [43]. In addition to this, the exogenous choline complex can be used as an exogenous source of choline that promotes the synthesis of acetylcholine. The combination of citicoline and cholinesterase inhibitors in the treatment of AD patients has been shown to be more effective in improving cognitive function and delaying disease progression than monotherapy using cholinesterase inhibitors [44, 45]. Surprisingly, we found that Cho levels in the basal forebrain were significantly higher than in the hippocampus, which suggests that the Cho peak is a potential cholinergic marker reflecting the cholinergic function of basal forebrain neurons. The decline in Cho/Cr ratio in the basal forebrain was observed in 6- and 8-month-old APP/PS1 mice compared with wild type mice of similar age, but we found no changes in the hippocampus. It is well recognized that the decline in the NAA/Cr ratio is a common manifestation of Alzheimer’s disease [23, 46]. Our findings support this conclusion as the NAA/Cr ratio in the basal forebrain and hippocampus decreased in APP/PS1 mice.

According to the “Cholinergic hypothesis of Alzheimer’s disease,” the loss of basal forebrain cholinergic neurons is likely behind characteristic cognition dysfunction of Alzheimer’s disease. By combining 3D reconstruction technology and immunohistochemistry (3D-IHC), a study found five familial AD mutations in 5XFAD mice associated with cholinergic-positive neuron loss in the basal forebrain at 9 months compared to age-matched C57BL/6 control mice. However, this was not observed in the 1st, 3rd, and 6th months of age [47]. We have previously shown that the degeneration of cholinergic neurons in the basal forebrain in an APP/PS1 animal model of AD occurred during the 7th month, including MS and VDB nuclei [48]. Another study showed that morphological changes of cholinergic fibers appeared in the cortex and hippocampus as early as 2–3 months of age in APP/PS1 mice. However, cholinergic neuron or ChAT activity remained unchanged in the forebrain structures with age [49]. Here, we applied Nissl staining to observe the arrangement and number of neurons present in the basal forebrain and hippocampus and found no significant reduction in 6-month-old APP/PS1 mice. In addition, we showed that neuron loss in the basal forebrain occurred at 8 months of age. The ChAT-specific staining results showed that the number of ChAT-positive in the MS and VDB nuclei in 6-month-old APP/PS1 mice was not decreased. However, the activity of ChAT enzyme is reduced. The “Amyloid cascade hypothesis” holds that Aβ-amyloid–containing plaques are the main cause behind the degeneration and death of peripheral neurons around senile plaques, as evidenced in numerous studies that detected Aβ deposition across many brain regions, including the cerebral cortex and the hippocampus [50, 51]. The results presented here are thus in accordance with previous observations. However, it is intriguing that we did not detect any Aβ plaques in the basal forebrain in 6-, 8-, and 13-month-old APP/PS1 mice, which suggests neuron degeneration in the basal forebrain is not associated with the presence of Aβ-amyloid–containing plaques. Several lines of evidence now support the idea that the dysfunction or even the loss of basal forebrain cholinergic neuron is associated with abnormal NGF signaling [52].

**Fig. 14:**
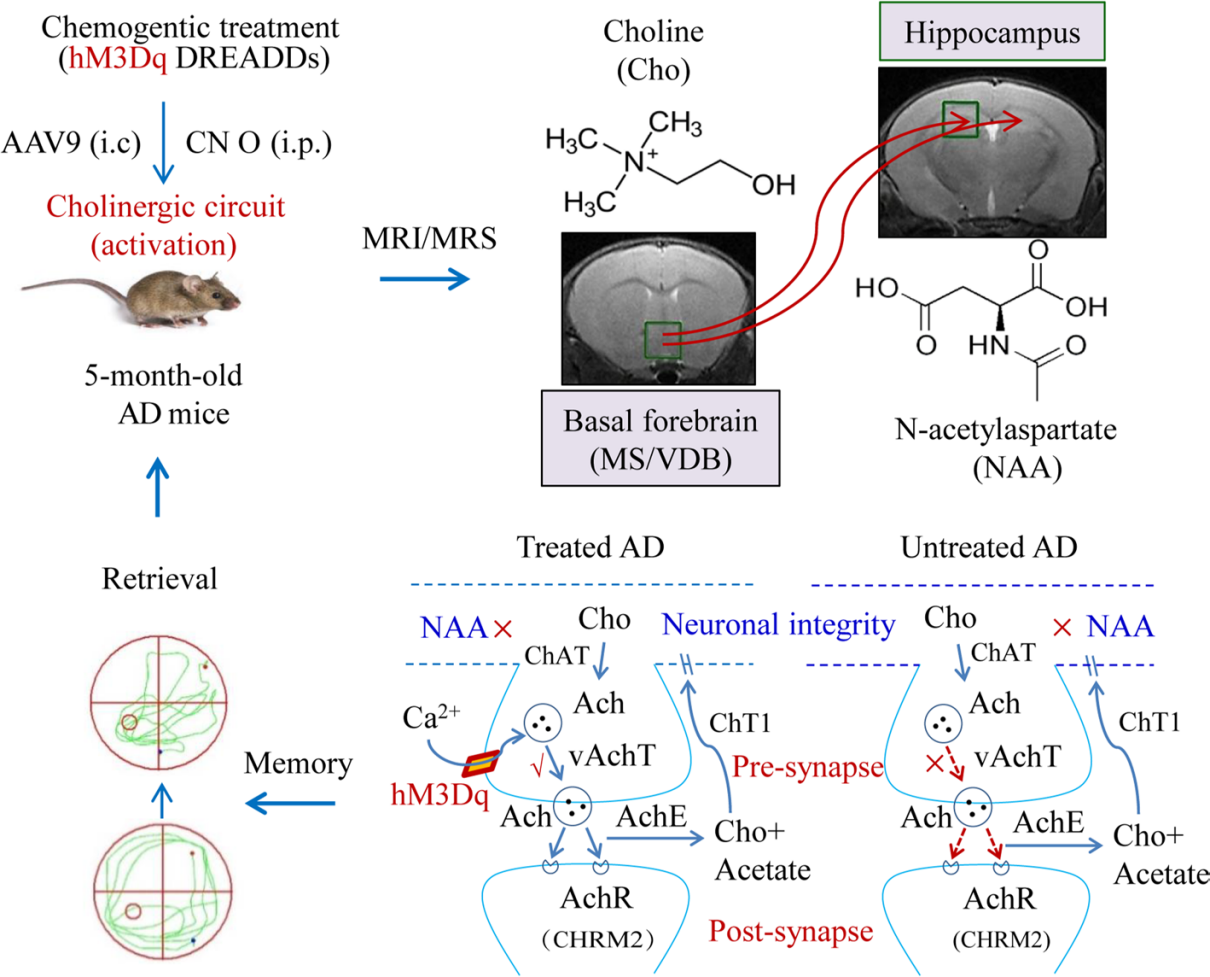
Activating the basal forebrain-hippocampal cholinergic neural circuit through chemical genetics might improve learning and memory in APP/PS1 mice by accelerating the choline cycle in the basal forebrain and the synthesis of NAA in the hippocampus

The disruption of cholinergic neurons in the basal forebrain leads to learning and memory deficits [53], while their activation through cholinesterase inhibitors, deep brain stimulation, and electrical stimulation technology seemingly improve cognitive function in Alzheimer’s disease [9, 54, 55]. However, these experimental approaches applied failed to achieve a precise regulation of cholinergic neurons in specific brain regions. Chemical genetic technology has been successfully applied in 5XFAD mice to reduce the deposition of Aβ plaque [56] and has been applied to improve cognitive function in AD rat models by stimulating noradrenergic neurons in the locus coeruleus [57]. We employed chemical genetic technology to regulate the cholinergic neural circuit from MS/VDB nuclei to the hippocampus in APP/PS1 mice. The behavioral tests suggest that the activation of the cholinergic neural circuit from MS/VDB nuclei to the hippocampus might significantly improve learning and memory abilities in APP/PS1 mice and is accompanied by elevated Cho/Cr and NAA/Cr ratios in the basal forebrain and hippocampus, respectively. NAA is mainly synthesized in the mitochondria of neurons, and a decrease in acetyl-CoA directly leads to NAA reduction, since the acetyl group of acetyl-CoA represents the main substrate for NAA synthesis [58]. Previous studies showed that cholinergic neurons exert far-reaching effects on the synthesis of NAA by regulating the activity of acetyl-CoA [59], whereby we hypothesize that chemical genetic technology is able to enhance the activity of cholinergic neurons in the basal forebrain in order to accelerate NAA synthesis. Similarly, donepezil treatment improves the cognitive function of patients with AD by increasing NAA levels in the hippocampus, cortex, and other brain regions [60, 61]. To confirm the activation of the cholinergic neural circuit from MS/VDB nuclei to the hippocampus, we evaluated the presence of cholinergic-related proteins in the basal forebrain and hippocampus. We found that the expression of ChAT and vAchT in the basal forebrain, and CHRM2 in the hippocampus, was enhanced after chemical genetic intervention. This suggests that chemical genetics triggers cascade activities including ACh transport, release, and synaptic transmission from the basal forebrain to the hippocampus, which can improve early memory impairment in APP/PS1 mice (Fig. 14). These findings might deepen our understanding of the cholinergic hypothesis in early AD stages and provide a basis for a variety of applications, such as chemogenetics, optogenetics, and deep brain stimulation (DBS) treatment of early AD.

### Limitations

Our research confirmed that chemical genetics technology activates the basal forebrain-hippocampus cholinergic neural circuit and can improve learning and memory in APP/PS1 mice. However, the underlying mechanisms need further study. For example, long-term potentiation represents the electrophysiological basis of learning and memory, whereby it is possible that chemical genetics technology improves learning and memory due to synaptic plasticity. Since electrophysiological recordings were not performed in this study, this hypothesis deserves further exploration in the future. It is worth noting that the pathological changes we found in APP/PS1 model mice do not represent all the pathological changes seen in human AD.

## Conclusion

Our study demonstrates that (1) cholinergic circuit Cho and NAA can be used as biomarkers for early AD and (2) chemical genetics-driven Ach cycle activities of the cholinergic circuit attenuate early memory impairment in APP/PS1 mice. Our findings help in clarifying the role of the cholinergic hypothesis in early AD.

## Supplementary Information

**Additional file 1: Figure S1.** No Aβ plaques were detected in the basal forebrain of APP/PS1 mice. (A, B) The deposition of Aβ plaques in APP/PS1 mice at the 8th month (A) and 13th month (B) and the boxed areas are shown below. (C, D) The higher magnification image of Aβ plaques in APP/PS1 mice at the 8th month (C) and 13th month (D).

**Additional file 2: Figure S2.** Learning and memory ability changes in AD mice by chemical genetics intervention. (A) DREADDs intervention timeline. (B, C) The 1h and 24h recognition index (RI) of the CNO and Saline groups after intervention. (D-F) The escape latency, platform crossing times and third quadrant time percentage of the CNO and Saline groups after intervention. *P<0.05.

## Data Availability

The data generated in this study is included in the published article.

## Abbreviations

3D-IHC: 3D reconstruction technology and immunohistochemistry
5XFAD: Five familial AD mutations
Ach: Acetylcholine
AchE: Acetylcholine esterase
AD: Alzheimer’s disease
AP: Anteroposterior
Aβ: Amyloid β
ChAT: Acetyltransferase
Cho: Choline
CHRM1: Muscarinic acetylcholine receptor 1
CHRM2: Muscarinic acetylcholine receptor 2
CNO: Clozapine-N-oxide
Cr: Creatine
DREADDs: Designer receptors exclusively activated by designer drugs
DV: Dorsoventral
FDG-PET: 18F-fluoro-2-deoxy-d-glucose positron emission tomography
hM3dq: Human muscarinic acetylcholine M3 receptor agonist
mI: Myo-inositol
ML: Mediolateral
MMSE: Mini-Mental State Examination
MRI: Magnetic resonance imaging
MRS: Magnetic resonance spectrum
MS: Medial septal nucleus
NAA: N-Acetyl aspartate
NOR: New object recognition
ppm: Part per million
RI: Recognition index
T2WI: T2-weighted imaging
TE: Echo time
TR: Repetition time
TF: Time that each mouse explores the familiar object
TN: Time that each mouse explores the unfamiliar object
vAchT: Vesicular acetylcholine transporter
VDB: Vertical limb of the diagonal band nucleus
VOI: Volumes of interest
WT: Wild type

## References

[1] Lian TH, Zhu WL, Li SW, Liu YO, Guo P, Zuo LJ, Hu Y, Yu SY, Li LX, Jin Z, Yu QJ, Wang RD, Zhang W (2019). Clinical, structural, and neuropathological features of olfactory dysfunction in patients with Alzheimer’s disease. J Alzheimers Dis.

[2] Talboom JS, Haberg A, De Both MD, Naymik MA, Schrauwen I, Lewis CR, et al. Family history of Alzheimer’s disease alters cognition and is modified by medical and genetic factors. Elife. 2019;8. 10.7554/eLife.46179 PMC6615857 31210642

[3] Sperling RA, Mormino EC, Schultz AP, Betensky RA, Papp KV, Amariglio RE, Hanseeuw BJ, Buckley R, Chhatwal J, Hedden T, Marshall GA, Quiroz YT, Donovan NJ, Jackson J, Gatchel JR, Rabin JS, Jacobs H, Yang HS, Properzi M, Kirn DR, Rentz DM, Johnson KA (2019). The impact of amyloid-beta and tau on prospective cognitive decline in older individuals. Ann Neurol.

[4] Blennow K, de Leon MJ, Zetterberg H (2006). Alzheimer’s disease. Lancet.

[5] Reinikainen KJ, Riekkinen PJ, Paljarvi L, Soininen H, Helkala EL, Jolkkonen J, Laakso M (1988). Cholinergic deficit in Alzheimer’s disease: a study based on CSF and autopsy data. Neurochem Res.

[6] Teipel SJ, Flatz WH, Heinsen H, Bokde AL, Schoenberg SO, Stockel S, Dietrich O, Reiser MF, Moller HJ, Hampel H (2005). Measurement of basal forebrain atrophy in Alzheimer’s disease using MRI. Brain.

[7] Grothe M, Heinsen H, Teipel S (2013). Longitudinal measures of cholinergic forebrain atrophy in the transition from healthy aging to Alzheimer’s disease. Neurobiol Aging.

[8] Teipel SJ, Meindl T, Grinberg L, Grothe M, Cantero JL, Reiser MF, Moller HJ, Heinsen H, Hampel H (2011). The cholinergic system in mild cognitive impairment and Alzheimer’s disease: an in vivo MRI and DTI study. Hum Brain Mapp.

[9] Hampel H, Mesulam MM, Cuello AC, Farlow MR, Giacobini E, Grossberg GT, Khachaturian AS, Vergallo A, Cavedo E, Snyder PJ, Khachaturian ZS (2018). The cholinergic system in the pathophysiology and treatment of Alzheimer’s disease. Brain.

[10] Bartus RT, Dean RR, Beer B, Lippa AS (1982). The cholinergic hypothesis of geriatric memory dysfunction. Science.

[11] Roman GC, Rogers SJ (2004). Donepezil: a clinical review of current and emerging indications. Expert Opin Pharmacother.

[12] Anand P, Singh B (2013). A review on cholinesterase inhibitors for Alzheimer’s disease. Arch Pharm Res.

[13] McShane R, Westby MJ, Roberts E, Minakaran N, Schneider L, Farrimond LE, Maayan N, Ware J, Debarros J (2019). Memantine for dementia. Cochrane Database Syst Rev.

[14] Loveman E, Green C, Kirby J, Takeda A, Picot J, Payne E, Clegg A (2006). The clinical and cost-effectiveness of donepezil, rivastigmine, galantamine and memantine for Alzheimer’s disease. Health Technol Assess.

[15] Ballinger EC, Ananth M, Talmage DA, Role LW (2016). Basal forebrain cholinergic circuits and signaling in cognition and cognitive decline. Neuron.

[16] Li X, Yu B, Sun Q, Zhang Y, Ren M, Zhang X, Li A, Yuan J, Madisen L, Luo Q, Zeng H, Gong H, Qiu Z (2018). Generation of a whole-brain atlas for the cholinergic system and mesoscopic projectome analysis of basal forebrain cholinergic neurons. Proc Natl Acad Sci U S A.

[17] Buzsaki G, Moser EI (2013). Memory, navigation and theta rhythm in the hippocampal-entorhinal system. Nat Neurosci.

[18] Gu Z, Alexander GM, Dudek SM, Yakel JL (2017). Hippocampus and entorhinal cortex recruit cholinergic and NMDA receptors separately to generate hippocampal theta oscillations. Cell Rep.

[19] Gu Z, Yakel JL (2011). Timing-dependent septal cholinergic induction of dynamic hippocampal synaptic plasticity. Neuron.

[20] Kantarci K, Jicha GA. Development of (1)H MRS biomarkers for tracking early predementia Alzheimer disease. Neurology. 2019. 10.1212/WNL.0000000000006839 30610092

[21] Watanabe T, Shiino A, Akiguchi I (2012). Hippocampal metabolites and memory performances in patients with amnestic mild cognitive impairment and Alzheimer’s disease. Neurobiol Learn Mem.

[22] Watanabe T, Shiino A, Akiguchi I (2008). Absolute quantification in proton magnetic resonance spectroscopy is superior to relative ratio to discriminate Alzheimer’s disease from Binswanger’s disease. Dement Geriatr Cogn Disord.

[23] Su L, Blamire AM, Watson R, He J, Hayes L, O'Brien JT (2016). Whole-brain patterns of (1)H-magnetic resonance spectroscopy imaging in Alzheimer’s disease and dementia with Lewy bodies. Transl Psychiatry.

[24] Liang S, Huang J, Liu W, Jin H, Li L, Zhang X, Nie B, Lin R, Tao J, Zhao S, Shan B, Chen L (2017). Magnetic resonance spectroscopy analysis of neurochemical changes in the atrophic hippocampus of APP/PS1 transgenic mice. Behav Brain Res.

[25] Sarter M, Lustig C, Howe WM, Gritton H, Berry AS (2014). Deterministic functions of cortical acetylcholine. Eur J Neurosci.

[26] Hasselmo ME, Sarter M (2011). Modes and models of forebrain cholinergic neuromodulation of cognition. Neuropsychopharmacol.

[27] Howe WM, Berry AS, Francois J, Gilmour G, Carp JM, Tricklebank M, Lustig C, Sarter M (2013). Prefrontal cholinergic mechanisms instigating shifts from monitoring for cues to cue-guided performance: converging electrochemical and fMRI evidence from rats and humans. J Neurosci.

[28] Mitsushima D, Sano A, Takahashi T (2013). A cholinergic trigger drives learning-induced plasticity at hippocampal synapses. Nat Commun.

[29] Pinto L, Goard MJ, Estandian D, Xu M, Kwan AC, Lee SH, Harrison TC, Feng G, Dan Y (2013). Fast modulation of visual perception by basal forebrain cholinergic neurons. Nat Neurosci.

[30] Sarter M, Lustig C, Blakely RD, Koshy CA (2016). Cholinergic genetics of visual attention: human and mouse choline transporter capacity variants influence distractibility. J Physiol Paris.

[31] Zhu H, Yan H, Tang N, Li X, Pang P, Li H, Chen W, Guo Y, Shu S, Cai Y, Pei L, Liu D, Luo MH, Man H, Tian Q, Mu Y, Zhu LQ, Lu Y (2017). Impairments of spatial memory in an Alzheimer’s disease model via degeneration of hippocampal cholinergic synapses. Nat Commun.

[32] Fink HA, Linskens EJ, MacDonald R, Silverman PC, McCarten JR, Talley K, Forte ML, Desai PJ, Nelson VA, Miller MA, Hemmy LS, Brasure M, Taylor BC, Ng W, Ouellette JM, Sheets KM, Wilt TJ, Butler M (2020). Benefits and harms of prescription drugs and supplements for treatment of clinical Alzheimer-type dementia. Ann Intern Med.

[33] Shen CJ, Zheng D, Li KX, Yang JM, Pan HQ, Yu XD, Fu JY, Zhu Y, Sun QX, Tang MY, Zhang Y, Sun P, Xie Y, Duan S, Hu H, Li XM (2019). Cannabinoid CB1 receptors in the amygdalar cholecystokinin glutamatergic afferents to nucleus accumbens modulate depressive-like behavior. Nat Med.

[34] Xiao L, Bornmann C, Hatstatt-Burkle L, Scheiffele P (2018). Regulation of striatal cells and goal-directed behavior by cerebellar outputs. Nat Commun.

[35] Miller BL (1991). A review of chemical issues in 1H NMR spectroscopy: N-acetyl-L-aspartate, creatine and choline. NMR Biomed.

[36] Zou J, Wang M, Lei X, Chen X (2014). 3.0T MRI arterial spin labeling and magnetic resonance spectroscopy technology in the application of Alzheimer’s disease. Exp Gerontol.

[37] Tumati S, Martens S, Aleman A (2013). Magnetic resonance spectroscopy in mild cognitive impairment: systematic review and meta-analysis. Neurosci Biobehav Rev.

[38] Griffith HR, den Hollander JA, Okonkwo OC, O'Brien T, Watts RL, Marson DC (2008). Brain metabolism differs in Alzheimer’s disease and Parkinson’s disease dementia. Alzheimers Dement.

[39] Kantarci K, Petersen RC, Boeve BF, Knopman DS, Tang-Wai DF, O'Brien PC, Weigand SD, Edland SD, Smith GE, Ivnik RJ, Ferman TJ, Tangalos EG, Jack CJ (2004). 1H MR spectroscopy in common dementias. Neurology.

[40] Kantarci K, Lowe V, Przybelski SA, Senjem ML, Weigand SD, Ivnik RJ, Roberts R, Geda YE, Boeve BF, Knopman DS, Petersen RC, Jack CJ (2011). Magnetic resonance spectroscopy, beta-amyloid load, and cognition in a population-based sample of cognitively normal older adults. Neurology.

[41] den Heijer T, Sijens PE, Prins ND, Hofman A, Koudstaal PJ, Oudkerk M, Breteler MM (2006). MR spectroscopy of brain white matter in the prediction of dementia. Neurology.

[42] Bittner DM, Heinze HJ, Kaufmann J (2013). Association of 1H-MR spectroscopy and cerebrospinal fluid biomarkers in Alzheimer’s disease: diverging behavior at three different brain regions. J Alzheimers Dis.

[43] Khomenko YG, Kataeva GV, Bogdan AA, Chernysheva EM, Susin DS (2019). Cerebral metabolism in patients with cognitive disorders: a combined MRS and PET study. Zh Nevrol Psikhiatr Im S S Korsakova.

[44] Castagna A, Cotroneo AM, Ruotolo G, Gareri P (2016). The CITIRIVAD Study: CITIcoline plus RIVAstigmine in Elderly Patients Affected with Dementia Study. Clin Drug Investig.

[45] Gareri P, Castagna A, Cotroneo AM, Putignano D, Conforti R, Santamaria F, Marino S, Putignano S (2017). The Citicholinage Study: Citicoline plus cholinesterase inhibitors in aged patients affected with Alzheimer’s disease study. J Alzheimers Dis.

[46] Piersson AD, Mohamad M, Rajab F, Suppiah S (2021). Cerebrospinal fluid amyloid beta, tau levels, apolipoprotein, and (1)H-MRS brain metabolites in Alzheimer’s disease: a systematic review. Acad Radiol.

[47] Yan H, Pang P, Chen W, Zhu H, Henok KA, Li H, Wu Z, Ke X, Wu J, Zhang T, Pan K, Pei L, Han Y, Lu Y (2018). The lesion analysis of cholinergic neurons in 5XFAD mouse model in the three-dimensional level of whole brain. Mol Neurobiol.

[48] Huang HJ, Liang KC, Ke HC, Chang YY, Hsieh-Li HM (2011). Long-term social isolation exacerbates the impairment of spatial working memory in APP/PS1 transgenic mice. Brain Res.

[49] Perez SE, Dar S, Ikonomovic MD, DeKosky ST, Mufson EJ (2007). Cholinergic forebrain degeneration in the APPswe/PS1DeltaE9 transgenic mouse. Neurobiol Dis.

[50] Wang X, Song R, Lu W, Liu Z, Wang L, Zhu X, Liu Y, Sun Z, Li J, Li X (2017). YXQN reduces Alzheimer’s disease-like pathology and cognitive decline in APPswePS1dE9 transgenic mice. Front Aging Neurosci.

[51] Wang J, Lu R, Yang J, Li H, He Z, Jing N, Wang X, Wang Y (2015). TRPC6 specifically interacts with APP to inhibit its cleavage by gamma-secretase and reduce Abeta production. Nat Commun.

[52] Pentz R, Iulita MF (2021). The NGF metabolic pathway: new opportunities for biomarker research and drug target discovery NGF pathway biomarkers and drug targets. Adv Exp Med Biol.

[53] Ramos-Rodriguez JJ, Pacheco-Herrero M, Thyssen D, Murillo-Carretero MI, Berrocoso E, Spires-Jones TL, Bacskai BJ, Garcia-Alloza M (2013). Rapid beta-amyloid deposition and cognitive impairment after cholinergic denervation in APP/PS1 mice. J Neuropathol Exp Neurol.

[54] Kuhn J, Hardenacke K, Shubina E, Lenartz D, Visser-Vandewalle V, Zilles K, Sturm V, Freund HJ (2015). Deep brain stimulation of the nucleus basalis of Meynert in early stage of Alzheimer’s dementia. Brain Stimul.

[55] Liu R, Crawford J, Callahan PM, Terry AJ, Constantinidis C, Blake DT (2017). Intermittent stimulation of the nucleus basalis of Meynert improves working memory in adult monkeys. Curr Biol.

[56] Yuan P, Grutzendler J (2016). Attenuation of beta-amyloid deposition and neurotoxicity by chemogenetic modulation of neural activity. J Neurosci.

[57] Rorabaugh JM, Chalermpalanupap T, Botz-Zapp CA, Fu VM, Lembeck NA, Cohen RM, Weinshenker D (2017). Chemogenetic locus coeruleus activation restores reversal learning in a rat model of Alzheimer's disease. Brain.

[58] Demougeot C, Marie C, Giroud M, Beley A (2004). N-acetylaspartate: a literature review of animal research on brain ischaemia. J Neurochem.

[59] Tomaszewicz M, Rossner S, Schliebs R, Cwikowska J, Szutowicz A (2003). Changes in cortical acetyl-CoA metabolism after selective basal forebrain cholinergic degeneration by 192IgG-saporin. J Neurochem.

[60] Jessen F, Traeber F, Freymann K, Maier W, Schild HH, Block W (2006). Treatment monitoring and response prediction with proton MR spectroscopy in AD. Neurology.

[61] Krishnan KR, Charles HC, Doraiswamy PM, Mintzer J, Weisler R, Yu X, Perdomo C, Ieni JR, Rogers S (2003). Randomized, placebo-controlled trial of the effects of donepezil on neuronal markers and hippocampal volumes in Alzheimer’s disease. Am J Psychiatry.

