# Supplementary figures and images for: Chemical genetic activation of the cholinergic basal forebrain hippocampal circuit rescues memory loss in Alzheimer’s disease
Source: Alzheimers Res Ther. 2022 Apr 13;14:53. doi: 10.1186/s13195-022-00994-w (PMC9006585; doi:10.1186/s13195-022-00994-w)

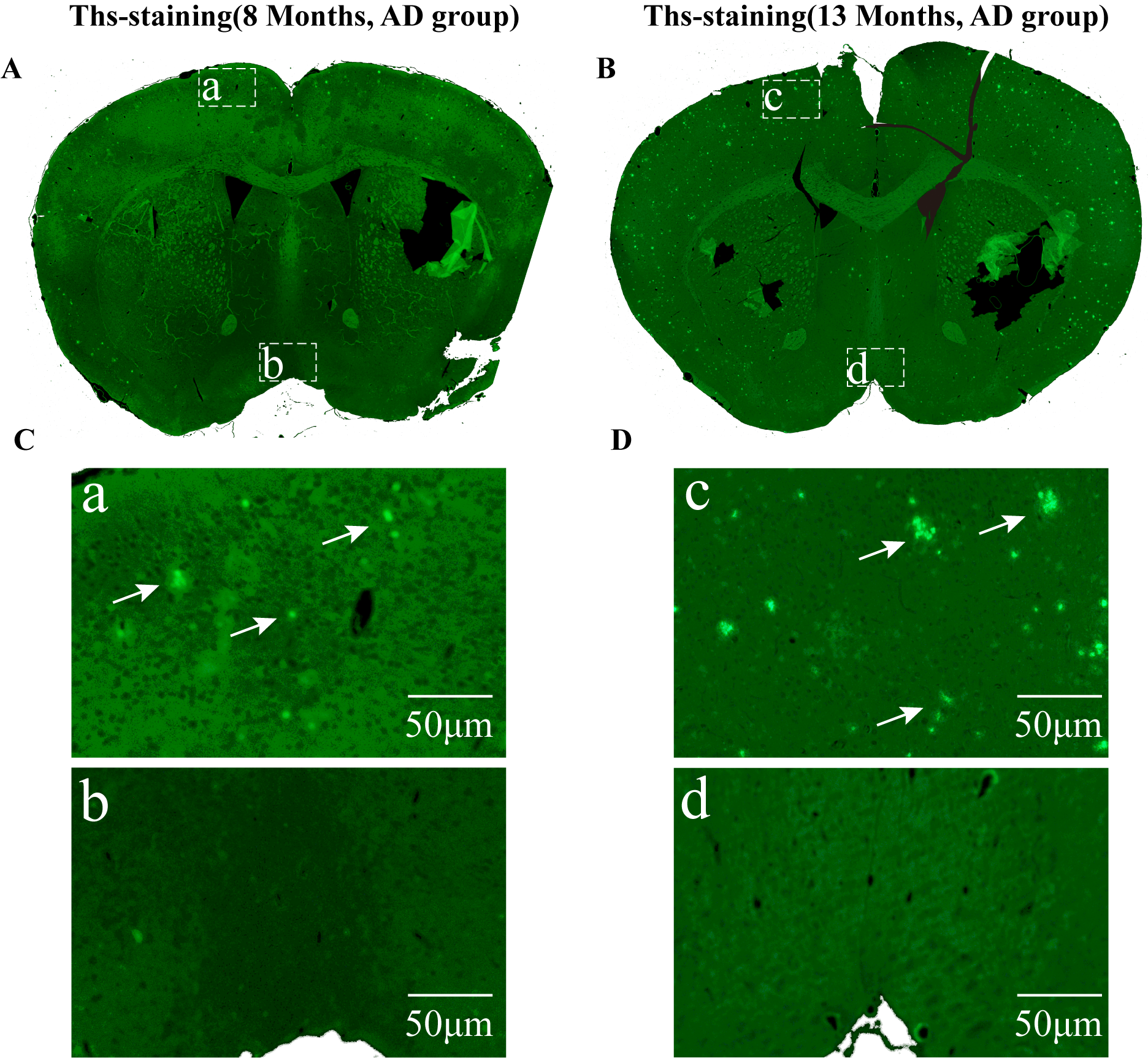

Supplement: Supplementary file 1 — Additional file 1: Figure S1. No Aβ plaques were detected in the basal forebrain of APP/PS1 mice. (A, B) The deposition of Aβ plaques in APP/PS1 mice at the 8th month (A) and 13th month (B) and the boxed areas are shown below. (C, D) The higher magnification image of Aβ plaques in APP/PS1 mice at the 8th month (C) and 13th month (D). [file 13195_2022_994_MOESM1_ESM.tif]

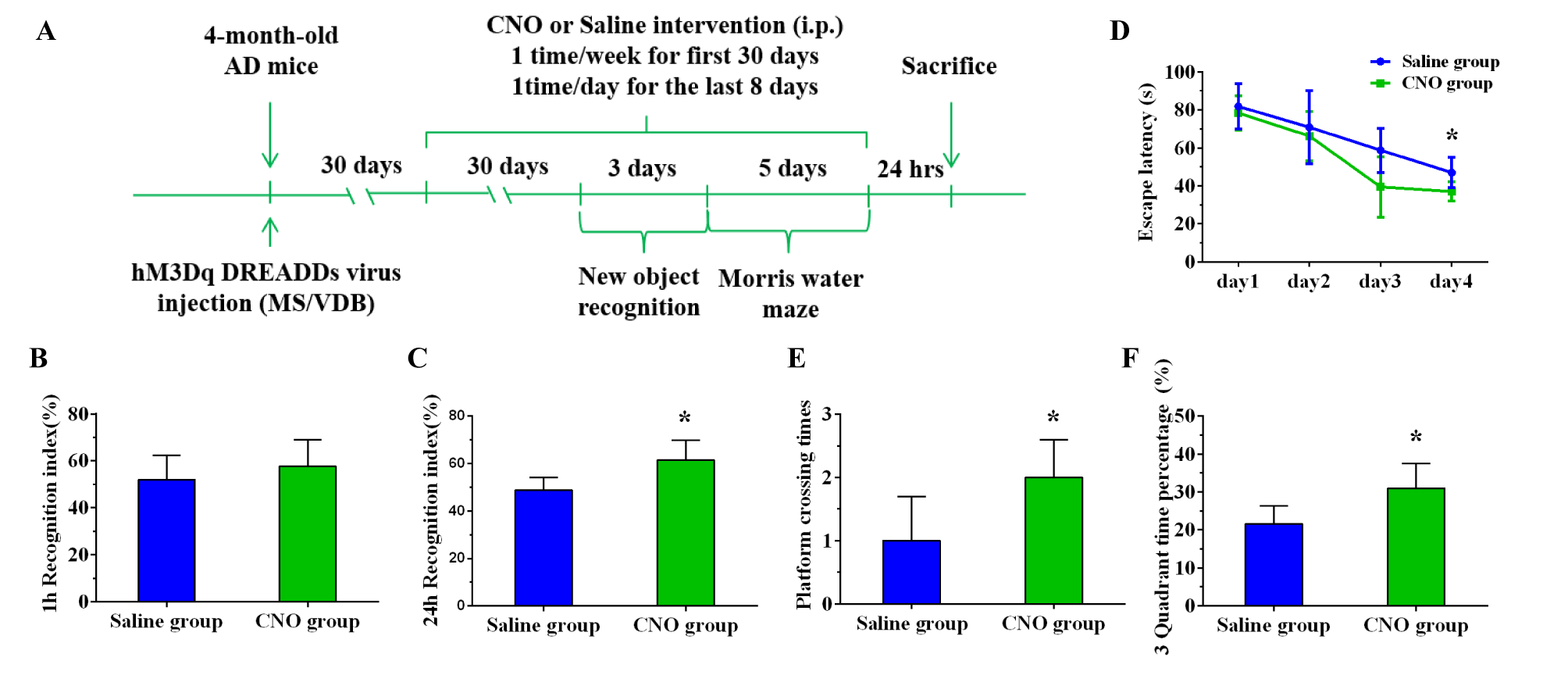

Supplement: Supplementary file 2 — Additional file 2: Figure S2. Learning and memory ability changes in AD mice by chemical genetics intervention. (A) DREADDs intervention timeline. (B, C) The 1h and 24h recognition index (RI) of the CNO and Saline groups after intervention. (D-F) The escape latency, platform crossing times and third quadrant time percentage of the CNO and Saline groups after intervention. *P<0.05. [file 13195_2022_994_MOESM2_ESM.tif]
